# Mycobacteria-Specific T Cells May Be Expanded From Healthy Donors and Are Near Absent in Primary Immunodeficiency Disorders

**DOI:** 10.3389/fimmu.2019.00621

**Published:** 2019-03-29

**Authors:** Shabnum Patel, Haili Lang, Gelina Sani, Alexandra F. Freeman, Jennifer Leiding, Patrick J. Hanley, Conrad Russell Cruz, Melanie Grant, Yunfei Wang, Benjamin Oshrine, Cindy Palmer, Steven M. Holland, Catherine M. Bollard, Michael D. Keller

**Affiliations:** ^1^Center for Cancer and Immunology Research, Children's National Health System, Washington, DC, United States; ^2^GW Cancer Center, George Washington University, Washington, DC, United States; ^3^Laboratory of Clinical Immunology and Microbiology, NIAID, National Institutes of Health, Bethesda, MD, United States; ^4^Division of Allergy & Immunology, University of South Florida, St. Petersburg, FL, United States; ^5^Department of Pediatrics, University of South Florida, St. Petersburg, FL, United States; ^6^Cancer and Blood Disorders Institute, Johns Hopkins All Children's Hospital, St Petersburg, FL, United States; ^7^Division of Blood and Marrow Transplantation, Children's National Health System, Washington, DC, United States; ^8^Clinical and Translational Science Institute, Children's National Health System, Washington, DC, United States; ^9^Division of Allergy & Immunology, Children's National Health System, Washington, DC, United States

**Keywords:** immunotherapy, T cells, mycobacteria, primary immunodeficiency, mendelian suspectibility to mycobacteria, hematopoietic stem cell transplantation

## Abstract

Mycobacterial Infections can be severe in patients with T-cell deficiency or phagocyte disorders, and treatment is frequently complicated by antimicrobial resistance. Restoration of T-cell immunity via stem cell transplantation facilitates control of mycobacterial infections, but presence of active infections during transplantation is associated with a higher risk of mortality. Adoptive T cell immunotherapy has been successful in targeting viruses, but has not been attempted to treat mycobacterial infections. We sought to expand and characterize mycobacterial-specific T-cells derived from healthy donors in order to determine suitability for adoptive immunotherapy. Mycobacteria-specific T-cells (MSTs) were generated from 10 healthy donors using a rapid *ex vivo* expansion protocol targeting five known mycobacterial target proteins (AG85B, PPE68, ESXA, ESXB, and ADK). MSTs were compared to T-cells expanded from the same donors using lysate from *M. tuberculosis* or purified protein derivative from *M. avium* (sensitin). MST expansion from seven patients with primary immunodeficiency disorders (PID) and two patients with IFN-γ autoantibodies and invasive *M. avium* infections. MSTs expanded from healthy donors recognized a median of 3 of 5 antigens, with production of IFN-γ, TNF, and GM-CSF in CD4^+^ T cells. Comparison of donors who received BCG vaccine (*n* = 6) to those who did not (*n* = 4) showed differential responses to PPE68 (*p* = 0.028) and ADK (*p* = 0.015) by IFN-γ ELISpot. MSTs expanded from lysate or sensitin also recognized multiple mycobacterial antigens, with a statistically significant differences noted only in the response to PPE68 (*p* = 0.016). MSTs expanded from patients with primary immunodeficiency (PID) and invasive mycobacterial infections showed activity against mycobacterial antigens in only two of seven subjects, whereas both patients with IFN-γ autoantibodies recognized mycobacterial antigens. Thus, MSTs can be generated from donors using a rapid expansion protocol regardless of history of BCG immunization. Most tested PID patients had no detectable T-cell immunity to mycobacteria despite history of infection. MSTs may have clinical utility for adoptive immunotherapy in T-cell deficient patients with invasive mycobacterial infections.

## Introduction

Mycobacteria species are ubiquitous, and mycobacterial infections account for over 1.5 million deaths annually (1). The global disease burden is skewed heavily toward tuberculosis, which infects ~1 in 3 people worldwide, but non-tuberculous mycobacteria are also a major cause of disease particularly in immunocompromised hosts (2, 3). Antibiotic resistance is especially common in non-tuberculous mycobacterial species, which often require long courses of multidrug treatments to combat infections (4).

Increased susceptibility to opportunistic infections is common in immunodeficient hosts, including individuals with primary immunodeficiency disorders (PID), transplant recipients, and patients receiving chemotherapy or immunosuppression for rheumatologic disease (5–7). It has been long appreciated that global T cell deficiency, such as occurs in severe combined immunodeficiency (SCID) and advanced HIV infection, is associated with risk of severe mycobacterial infections. Invasive infections following vaccination with *Bacillus Calmette Guerin* (BCG) has unfortunately remained a common presenting sign in infants with SCID (2, 3). In the past decade, many essential immunologic pathways that mediate control of mycobacterial infections have been described, grouped together as Mendelian Susceptibility to Mycobacterial Disease (MSMD) (8). MSMD can result from deficiencies in the IL-12/IFN-γ pathway, ISG15, and signaling pathways downstream of IFN-γ including STAT1, the CBM/IkB-kinase complex, and the transcription factor NF-kB (9–13). Developmental defects in myeloid cells caused by mutations in *IRF8* or *GATA2* also result in MSMD (14, 15).

Immunologic responses to mycobacterial antigens have been well-described, and delayed type hypersensitivity to tuberculosis antigens is utilized for clinical testing for tuberculosis exposure via IFN-γ ELISpot assay (16, 17). Anergy on tuberculosis testing has also been well-documented in patients with T cell immunodeficiencies, even in the presence of mycobacterial infections (18). Restoration of T cell immunity via antiretroviral therapy in the setting of HIV substantially reduces the risk of invasive mycobacterial infections (19). In PID however, the presence of an invasive mycobacterial infection may significantly worsen the risks of transplantation (20, 21).

In the setting of hematopoietic stem cell transplantation, adoptive immunotherapy with virus-specific T cells (VST) has been utilized for over two decades with strong evidence of safety and efficacy (22–24). Recent efforts have heavily focused on the use of “third party” banks of well-characterized VSTs derived from healthy donors, which can be used as partially HLA-matched, “off the shelf” therapies for the treatment of viral infections (25–28). Though matching algorithms for the use of these products are evolving, the success rate for partially HLA-matched VSTs has improved. In several cases, VSTs have been utilized successfully for the treatment of viral infections prior to HSCT in children with severe PIDs (29, 30). Though mycobacteria are much more complex organisms than the viruses targeted in previous adoptive immunotherapy trials, many immunodominant mycobacterial T-cell antigens have been described (17, 31). Hence, adoptive immunotherapy targeting mycobacterial antigens may be similarly beneficial as a therapeutic strategy to control invasive mycobacterial infections before, during or after HSCT.

In this study, we demonstrate that T cells targeting mycobacterial antigens can be robustly expanded from healthy donors using a protocol that is compatible with Good Manufacturing Practices (32). Many of the targeted epitopes are conserved across species, allowing cross-reactivity against different mycobacteria. We also demonstrate decreased to absent T cell responses against these antigens as a consistent feature in patients with PIDs with invasive mycobacterial infections.

## Methods

### Subjects and Patients

Healthy donors and patients were consented on research protocols for blood donation at Children's National Medical Center, the National Institutes of Health, and All Children's Hospital. Donors were evaluated for prior history of BCG vaccination, and those who were vaccinated were evaluated for recent histories of positivity on tuberculin or Quantiferon testing. Patient samples were obtained from individuals with primary immunodeficiency disorders and the presence of an active or recent invasive infection with *M. avium* complex or *M. abscessus* (Supplemental Table 1). All research protocols were approved by the Institutional Review Boards at the host institutions.

### Isolation of Peripheral Blood Mononuclear cells

Peripheral blood mononuclear cells (PBMCs) were isolated via Ficoll density centrifugation. Blood was diluted 1:1 in phosphate buffered saline, layered on top of 10–15 mL of Lymphocyte Separation Medium (MP Biomedicals, CA), and spun for 40 min at 400 G at room temperature. PBMCs were harvested from the lymphocyte layer and washed twice with 1X PBS prior to counting and generation of MST lines.

### Rapid Generation of Mycobacteria-Specific T Cells From Healthy Donors and Patients

**Figure 1:**
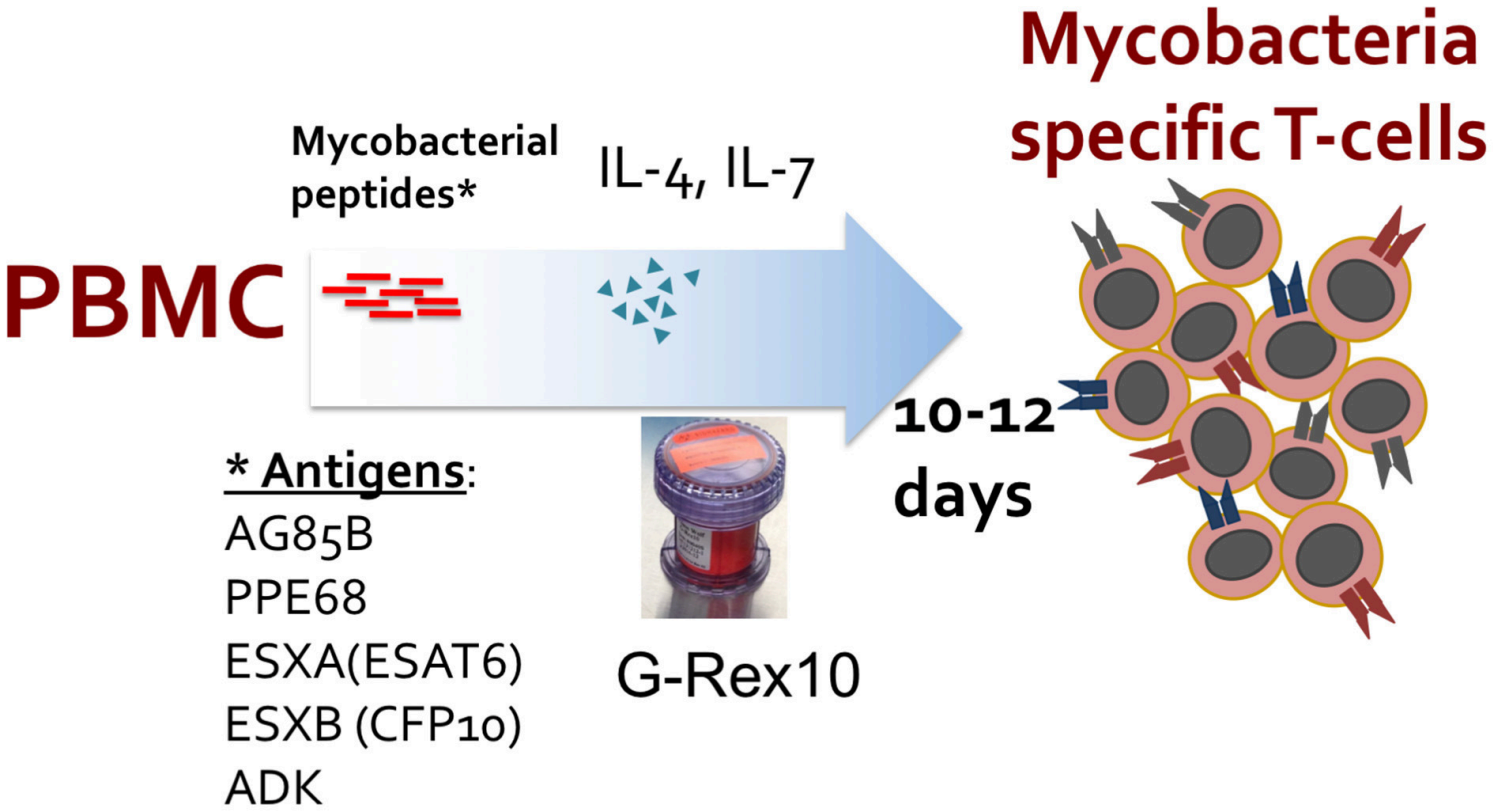
Manufacturing schema of *ex vivo* expansion of mycobacteria-specific T cells. Peripheral blood mononuclear cells (PBMCs) are stimulated with overlapping peptide pools encompassing listed mycobacterial antigens and cultured in a G-Rex-10 bioreactor with cytokines for 10–12 days.

On Day 1, PBMCs (10–15 × 10^6^) were pelleted in a 50 ml conical tube. Overlapping 15-mer peptide pools encompassing antigens from *M. tuberculosis* (pepmixes) were pooled, with 2 μl of each TB pepmix (five 15-mer pepmix libraries, each reconstituted at a concentration of 0.5 nmol/μL) added to 200 μl CTL medium (45% RPMI, 45% Click's medium, 10% fetal bovine serum with 2 mmol L-glutamine), with a final peptide concentration of 25 nmol/ml. TB pepmixes included peptides from AG85B, PPE68 (Rv3873), ESXA (ESAT-6), ESXB (CFP-10), and ADK. Protein consensus sequences were obtained from NCBI RefSeq (Supplemental Table 2) for pepmix generation (JPT, Berlin, Germany). PBMC pellets were resuspended in 200 μl of the CTL medium/pepmix and incubated at 37°C for 30–60 min (Figure 1). After incubation, PBMCs were resuspended in CTL medium/10% FBS with IL-7 (10 ng/ml) and IL-4 (400 U/ml) at a final concentration of 1 × 10^6^ cells/ml (R&D Systems, MN). Pepmix-pulsed PBMCs were plated in 24-well plates at 2 ml/well. On Days 3–5, culture medium was monitored for color and cell confluence. For confluent cultures, half-medium change (with IL-7 and IL-4) was performed. On Day 7, culture medium was monitored again and cells were split 1:1 if confluent with a half-medium change. On Days 10–12, cells were harvested and evaluated for antigen specificity and functionality.

### MST Generation From Healthy Donors With *M. Avium* Sensitin or TB Lysate

*M. tuberculosis* lysate (Strain CDC1551, BEI Resources, Manassas, VA) was reconstituted in 10 mM ammonium bicarbonate at 10 mg/ml. *M. avium* Sensitin (Statens Serum Institut, Denmark, provided courtesy of Dr. Ford von Reyn, Dartmouth University) protein was reconstituted at 1 ug/ml in 1.5 ml of saline. On Day 1, PBMCs (10–15 × 10^6^) were co-incubated with lysates at the following conditions: *M. avium* Sensitin (50 ng) or *M. tuberculosis* lysate (100 μg). PBMCs + lysates were resuspended in CTL medium/10% FBS with IL-7 (10 ng/ml) and IL-4 (400 U/ml) at a final concentration of 1 × 10^6^ cells/ml and plated in 24-well plates at 2 ml/well. On Days 3–7, culture medium was monitored as before and changed as appropriate. On Days 10–12, cells were harvested and evaluated for TB-specificity and functionality.

### IFN-γ ELISPOT Assay and Epitope Mapping

Antigen specificity of T cells was measured with IFN-γ ELISPOT (Millipore, Burlington, MA). T cells were plated at 1 × 10^5^/well with no peptide or actin (negative controls), *Staphylococcus* enterotoxin B (SEB) (positive control), or TB pepmix and lysate as stimulants. Specificity was defined as a minimum of 20 spot forming cells (SFC)/1 × 10^5^ cells/well with statistical significance of the result over the negative controls by two-tailed Student's *T*-Test (*p* < 0.05). For epitope mapping, 15 mer peptides were synthesized (GenScript, Piscataway Township, NJ, USA) which spanned the entire AG85B and ESXB proteins, with overlaps of five amino acids between each peptide. ELISPOT plates were sent for IFN-γ SFC counting and confluence determination (Zellnet Consulting, Fort Lee, NJ, USA).

### Immunophenotyping of MSTs

Phenotyping of MST cell cultures was performed by flow cytometry with antibodies against CD3, CD8, CD4, CD25, CD14, CD16, CD19, CD27, CD28, CD45RA, CD45RO, CD56, CD57, CD62L, CD127, CCR7, IFN-γ, TNF, CD223 (LAG3), CD95, Perforin, PD-1, TCRγδ, CTLA4, and TIM3 (Milenyi Biotec, Bergisch Gadbach, Germany; Biolegend, San Diego, CA, USA; BD Bioscience, San Jose, CA, USA; Invitrogen, Carlsbad, CA, USA; and Ebioscience, San Diego, CA, USA) (Supplemental Table 3). On Day 1, MSTs from healthy and BCG-vaccinated donors were rested overnight with low dose IL-2 (50 U/mL). On Day 2, T cells were washed and plated at 1 × 10^6^ cells/well with corresponding pepmix, αCD28/CD49 co-stimulator, and Brefeldin A and incubated for 6 h. Conditions were as follows: no pepmix, actin pepmix, SEB, or a mix of mycobacterial peptides (equal concentrations of PPE68, ESXA, ESXB, AG85B, and ADK pepmixes) at 2.5 ug/well. After 6 h incubation in the above conditions, cells were washed, stained for surface markers, washed, and fixed with 4% paraformaldehyde. Cells were then permeabilized with saponin (Perm Wash Buffer, BD Biosciences, San Diego, CA), stained with intracellular antibodies, and washed. T cells transduced with a chimeric antigen receptor specific for GD2 were utilized as a control for presence of co-inhibitory receptors (courtesy of Dr. Crystal Mackall, Stanford University) (33). Samples were acquired on a CytoFlex S Flow Cytometer (Beckman Coulter, Indianapolis, IN, USA), and analyzed in FlowJo VX (FlowJo LLC, Ashland, OR, USA). Standardized gating strategies were utilized for surface staining (Supplemental Figure 1) and intracellular staining (Supplemental Figure 2).

### Multiplex Cytokine Assay

MST product functionality was measured with the Bioplex Pro Human 17-plex Cytokine Assay kit (Biorad, Hercules, CA, USA). On Day 1, MSTs from healthy donors were rested overnight with low dose IL-2 (50 U/mL). On Day 2, T cells were washed and plated at 1 × 10^6^ cells/well with 1 μl of corresponding pepmix. Conditions were as follows: No pepmix (control), actin only (control), SEB (positive control), AG85B, PPE68, ESXA, ESXB, or ADK at 1 ug/well. On Day 3, supernatants were harvested from the wells, spun down to remove debris, and plated on the multiplex plate. For immunodeficient patients, supernatants were collected from ELISPOT plates to be run on 17-plex, due to limited cell numbers. The Biorad 17-plex multiplex manufacturer's protocol was followed and read on a MAGPIX System (Luminex, Austin, TX).

### HLA Typing

Selected donor samples were sent for high resolution SSO HLA typing (Kashi Clinical Laboratories, Portland, OR).

### Data Analysis

Data analysis was performed in Graphpad Prism (GraphPad Software, La Jolla, CA) and SAS 9.3 (SAS Institute, Cary, NC). The Kruskal-Wallis test with two-tail significance level α of 0.05 was used to test for differences between multiple data groups, and two-tailed *T*-tests were used for pairwise data analysis.

## Results

### Mycobacteria-Specific T Cells Can Be Expanded From Healthy Donors

Ten healthy donors were evaluated for T cell responses to mycobacterial antigens. Six donors had prior histories of BCG vaccination, of whom three had known histories of positivity on delayed type hypersensitivity testing but negative chest radiographs. One had a previously negative Quantiferon test.

**Figure 2:**
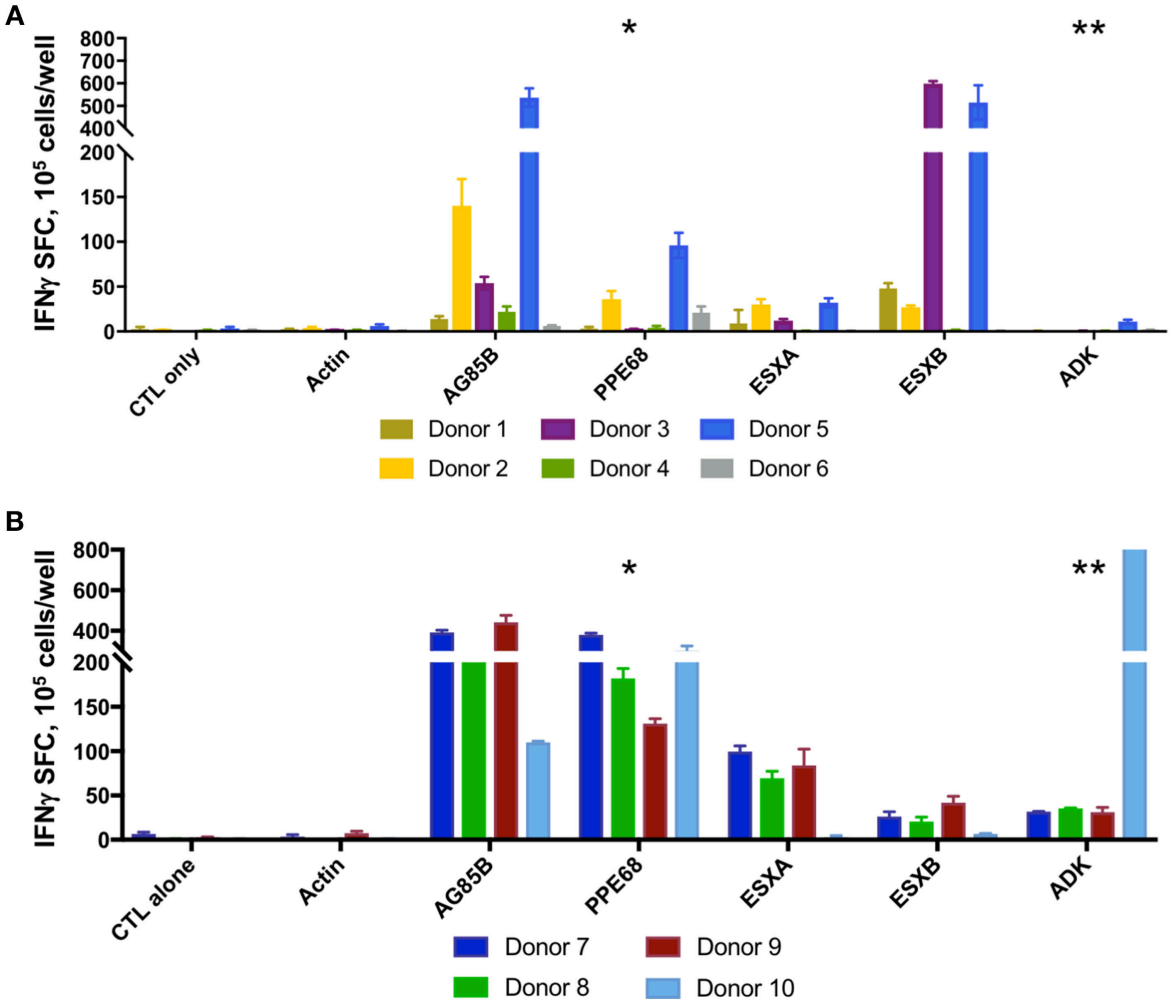
MSTs expanded from healthy donors recognize multiple mycobacterial antigens. IFN-γ ELISpot of *ex vivo* expanded MSTs at day 10 showed specificity to multiple mycobacterial antigens in both BCG immunized donors **(A)** and non-BCG vaccinated donors **(B)**. Significant differences between groups was noted in the responses against PPE68 (*p = 0.028) and ADK (**p = 0.015). SFC, Spot forming colonies.

**Figure 3:**
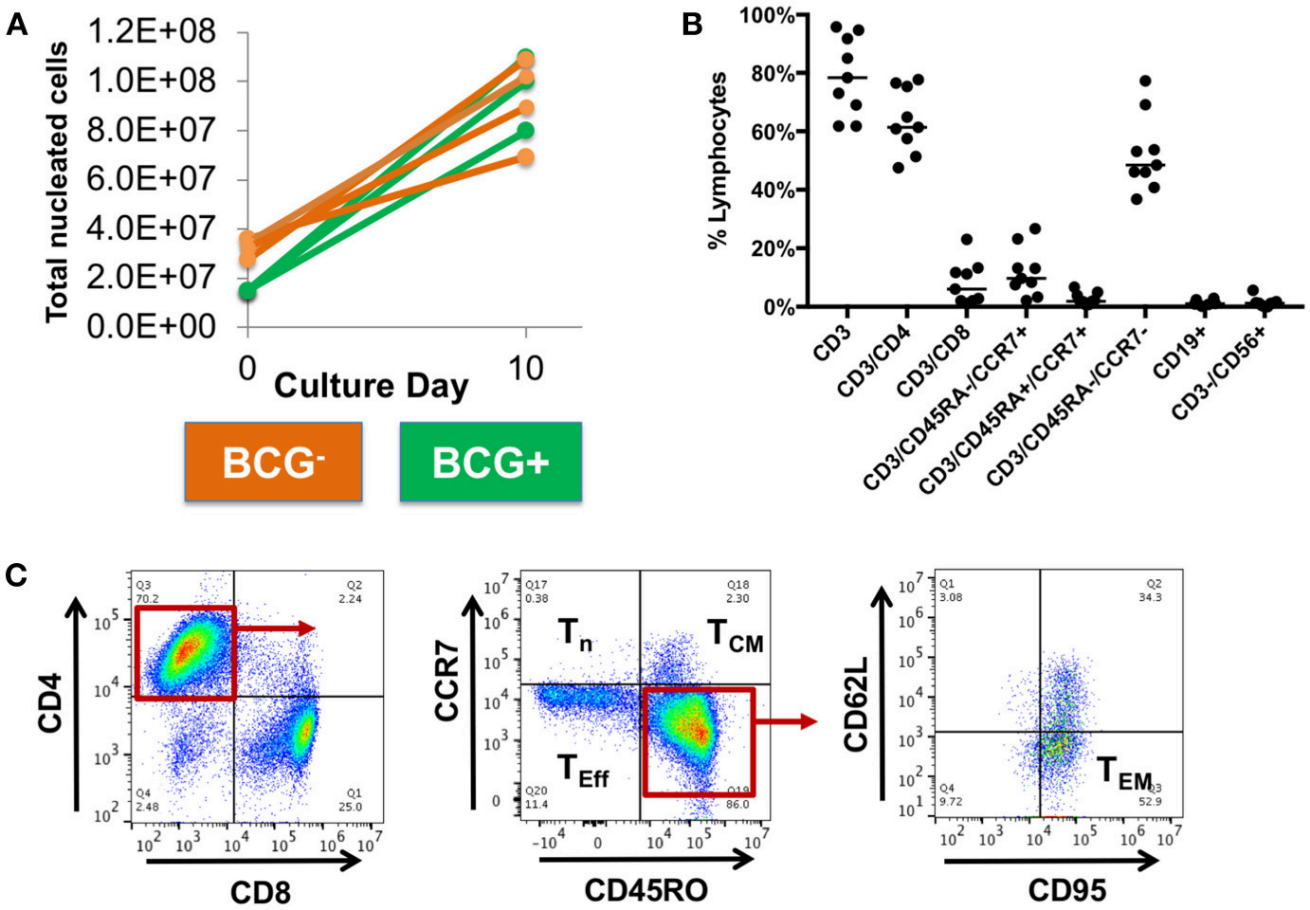
Expanded MSTs are mostly CD4+ effectors. **(A)** Mycobacterial-specific T cells expanded during culture with a mean fold-expansion of 4.4. BCG- = BCG non-immunized; BCG^+^ = BCG immunized. **(B)** Surface phenotyping of MSTs following expansion showed a predominance of CD4+ T cells with large effector memory population and smaller central memory population. Lines, median value. **(C)** Example plots from MSTs expanded from Donor 9 show a large CD4^+^ effector memory (T_EM_) population and smaller effector (T_eff_) and central memory (T_cm_) population, with minimal naïve T cells (T_n_).

Following 10-days *ex vivo* expansion of MSTs, IFN-γ ELISpot demonstrated reactivity against a median of 3 of 5 antigens per subject (range 1–5, Supplemental Table 4). Comparison of BCG-immunized (Figure 2A) and non-immunized donors (Figure 2B) demonstrated a greater likelihood of response to PPE68 (*p* = 0.028) and ADK (*p* = 0.015) in the BCG-non-immunized donors (Supplemental Table 5). Cultures underwent a mean 4.4-fold expansion, with recovery of 7–11 × 10^7^ cells (Figure 3A).

### *Ex vivo* Expanded MSTs Are Predominantly CD4^+^ T Cells

Flow cytometry of bulk MSTs following culture showed that the majority of cells were CD4^+^ T cells (median 63.7% CD3^+^/CD4^+^, range 47.5–77.7%, Figure 3B), with a small minority of CD8^+^ T cells (median 6% CD3^+^/CD8^+^, range 1.1–23%). The majority of CD4^+^ T cells were effectors (median 66.1%, range 60.8–68.9%) with a smaller central memory population (median 1.6%, range 1.4–4.4%) (Figure 3C). There was no outgrowth of B cells or NK cells.

**Figure 4:**
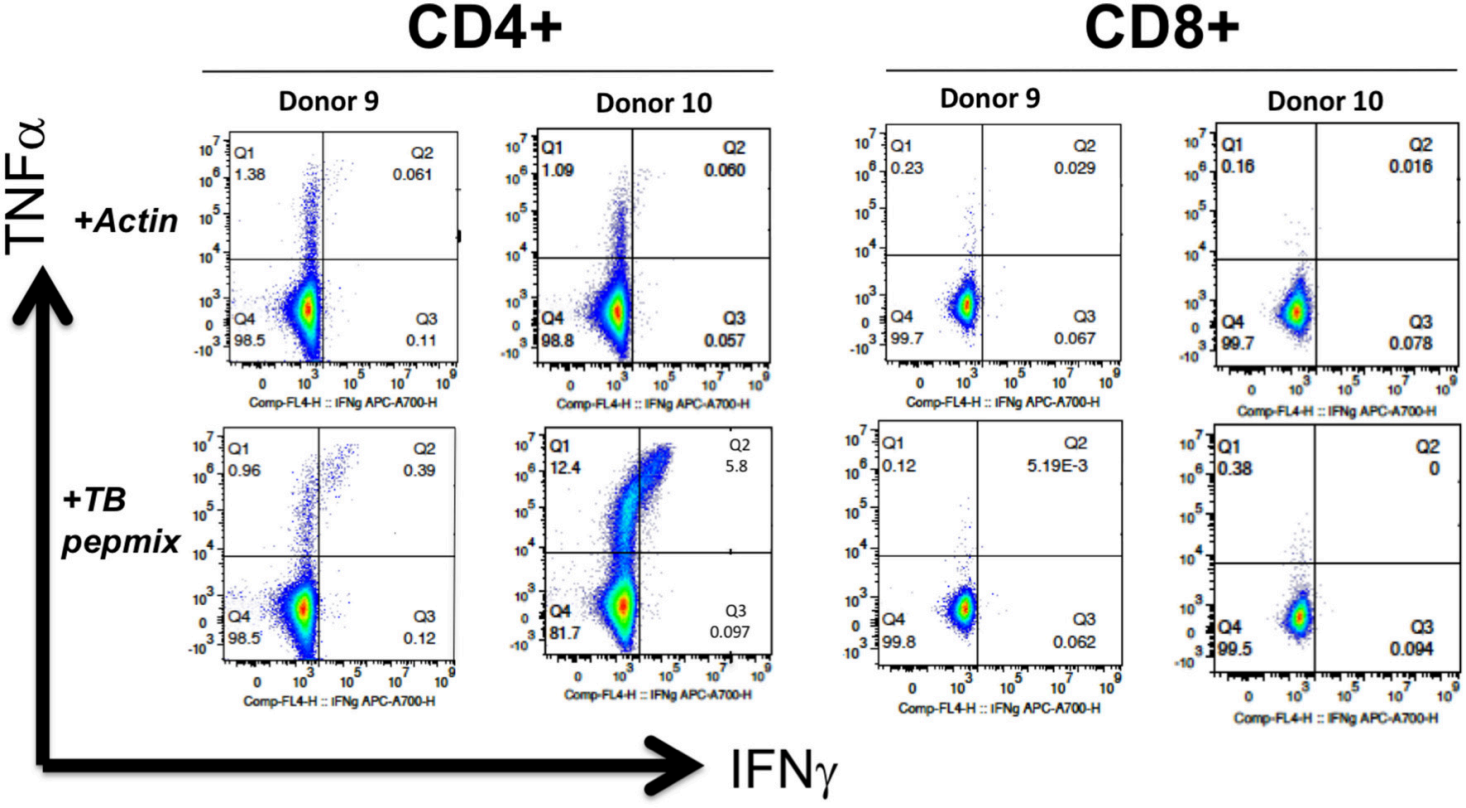
MSTs expanded from healthy donors are polyfunctional. Intracellular flow cytometry demonstrated production of IFN-γ and TNF in response to mycobacterial pepmix restimulation exclusively in CD4^+^ T cells from MSTs expanded from healthy donors, with no responses seen in CD8^+^ T cells.

Upon restimulation with mycobacterial pepmix, CD4^+^ T cells from most donors showed polyfunctionality with production of TNF and IFN-γ (Figure 4). Multiplex cytokine analysis also showed production of IL-8, IL-10, IL-13, GM-CSF, MCP1, and MIP-1b. Most of these cytokines were present at baseline, with only IFN-γ, TNF, and GM-CSF showing increases in response to peptide restimulation in all tested healthy donors (*n* = 4), vs. IL-13 (2 of 4) or MIP-1b (3 of 4) (Supplemental Figure 3). Minor CD8^+^ T-cell fractions expressed perforin at baseline (Supplemental Figure 4), but had minimal antigen-specific cytokine release. Expanded MSTs showed low expression of PD1, low to moderate surface expression of inhibitory co-receptor LAG3, but lower TIM3 expression than the positive control GD2-CAR T cells, which are known to highly express co-inhibitory receptors (Supplemental Figure 5).

### Mycobacterial Responses Are Largely Absent in Patients With Primary Immunodeficiency

**Figure 5:**
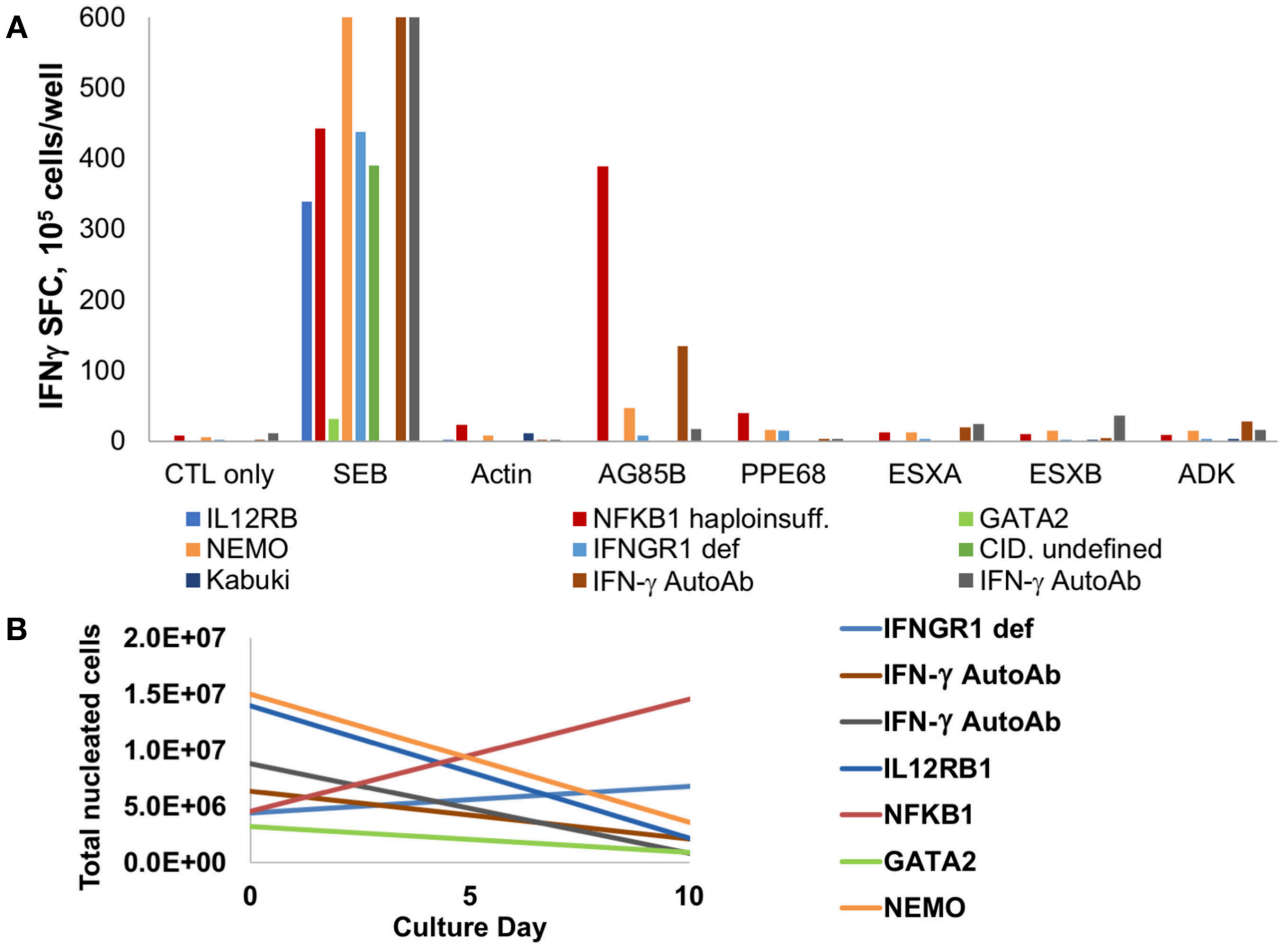
MSTs are deficient in most patients with PID. **(A)** IFN-γ ELISpot of T cells expanded from patients with primary immunodeficiency disorders (PID) showed decreased to absent responses to mycobacterial antigens, with exception of a patient with NFKB1 haploinsufficiency. Two patients with IFN-γ autoantibodies had detectable responses. SEB, staphylococcal enterotoxin B; CID, combined immunodeficiency. **(B)** *Ex vivo* culture of T cells from patients with PID yielded no expansion in all but two patients.

Seven subjects with primary immunodeficiency disorders and invasive infections with *M. avium* complex or *M. abscessus* were tested for responses against mycobacterial antigens. Underlying diagnoses were IL12RB1 deficiency, NFKB1 haploinsufficiency, IFNGR1 deficiency, GATA2 haploinsufficiency, Kabuki syndrome, NEMO deficiency, and undefined combined immunodeficiency (CID). Two patients with anti-IFN-γ autoantibodies and invasive infections with *M. avium* and *M. abscessus* were also evaluated. Following a 10-day expansion, evaluation of specificity via IFN-γ ELISPOT demonstrated specificity to mycobacterial antigens in two of the seven patients with PID (Figure 5A, Supplemental Table 6). The subject with NFKB1 haploinsufficiency had robust IFN-γ production to AG85B on ELISPOT and a lesser response to PPE68, and a subject with NEMO had low-level response to AG85B. Further evaluation of T cells from the NFKB1 patient showed no evidence of T cell exhaustion based on expression of inhibitory co-receptors (Supplemental Figure 5). Studies of the T cells of three PID subjects via multiplex cytokine analysis showed no cytokine production in response to mycobacterial pepmix in two patients, and isolated production of IL-8 in a subject with IL12RB1 deficiency (Supplemental Figure 3). Both of the subjects with anti-IFN-γ autoantibodies had detectable T cell responses to mycobacterial antigens (AG85B and ADK in one subject, and ESXA and ESXB in the other). Cell expansion during the culture period was minimal or absent in all patients (Figure 5B) with the exception of the subject with NFKB1 haploinsufficiency (3.2-fold expansion).

### MSTs Expanded Against *M. tuberculosis* Lysate or *M. avium* Sensitin Recognize Immunodominant Antigens

**Figure 6:**
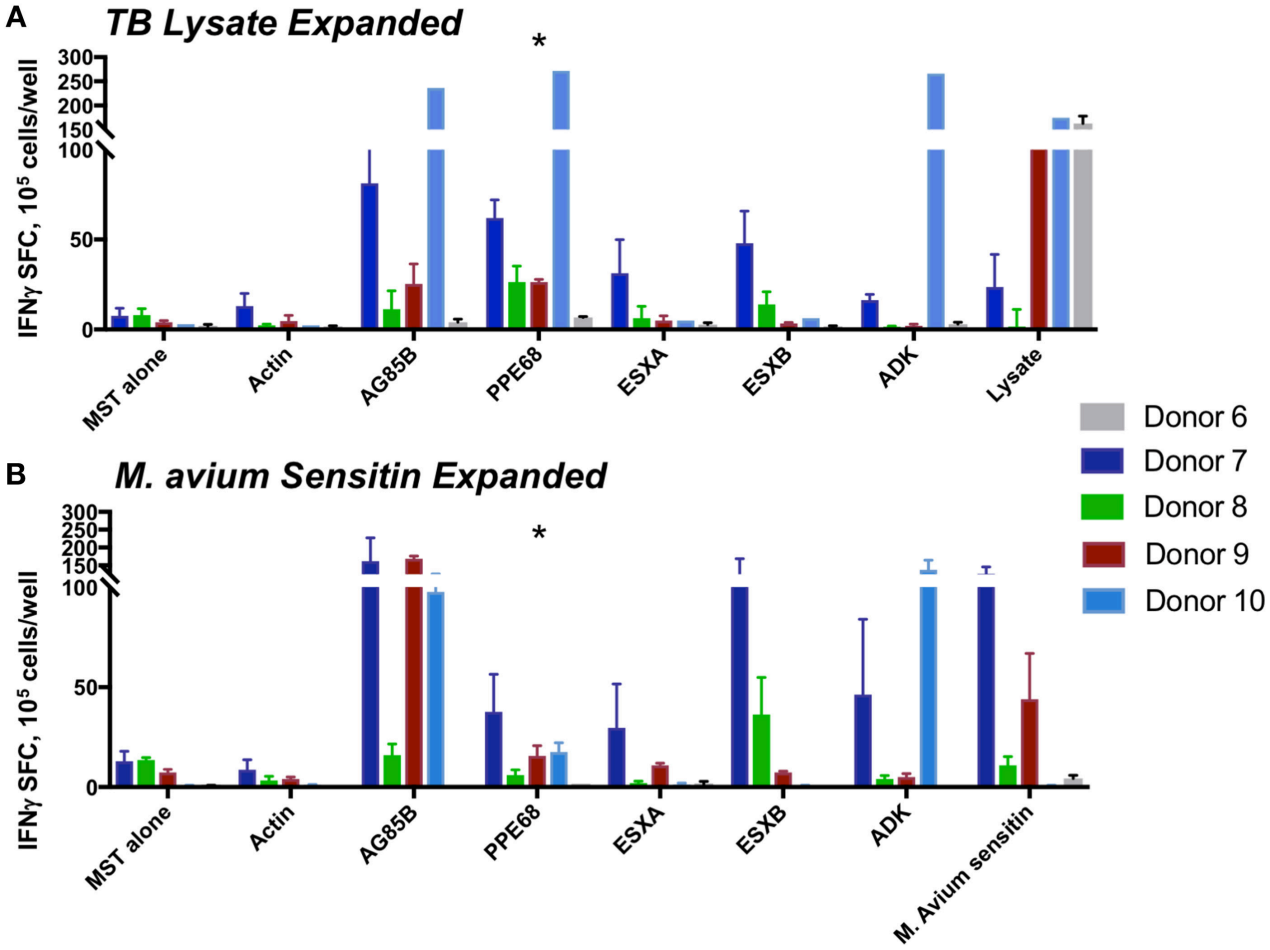
MST responses are comparable using peptide stimulation vs. lysate or sensitin. IFN-γ ELISpot from MSTs expanded using TB lysate **(A)** or *M avium* sensitin **(B)** showed specificity to multiple mycobacterial pepmixes, which were comparable in magnitude to the response to restimulation with lysate or sensitin. Differences in responses were only significant for PPE68 (*p = 0.032). SFC, spot forming colonies; SEB, staphylococcal enterotoxin B.

Following 10 days of culture after stimulation with lysate from *M. tuberculosis* or M*. avium* sensitin, MSTs from all five tested donors showed specificity for the mycobacterial antigen pepmixes or against lysate or sensitin (Figure 6). Analysis of MST responses via IFN-γ ELISPOT following expansion against the pepmixes, lysate, or sensitin showed significant differences in the response to PPE68 (*p* = 0.032), but not to the other antigens (Supplemental Table 7). Pairwise analysis showed a statistically significant difference in the response to PPE68 of MSTs generated using pepmix vs. sensitin (*p* = 0.016), but no difference between MSTs generated using pepmix vs. lysate (*p* = 0.173) or lysate vs. sensitin (*p* = 0.116). Comparative surface flow cytometry of MSTs generated using pepmix, sensitin, or lysate, all showed a predominance of CD4^+^ effector memory cells, with no notable differences between subpopulations, and a minimal percentage of γ/δ T-cells (Supplemental Figure 6).

### Epitopes in Mycobacterial AG85B and ESXB Are Variably Conserved Across Species

**Figure 7:**
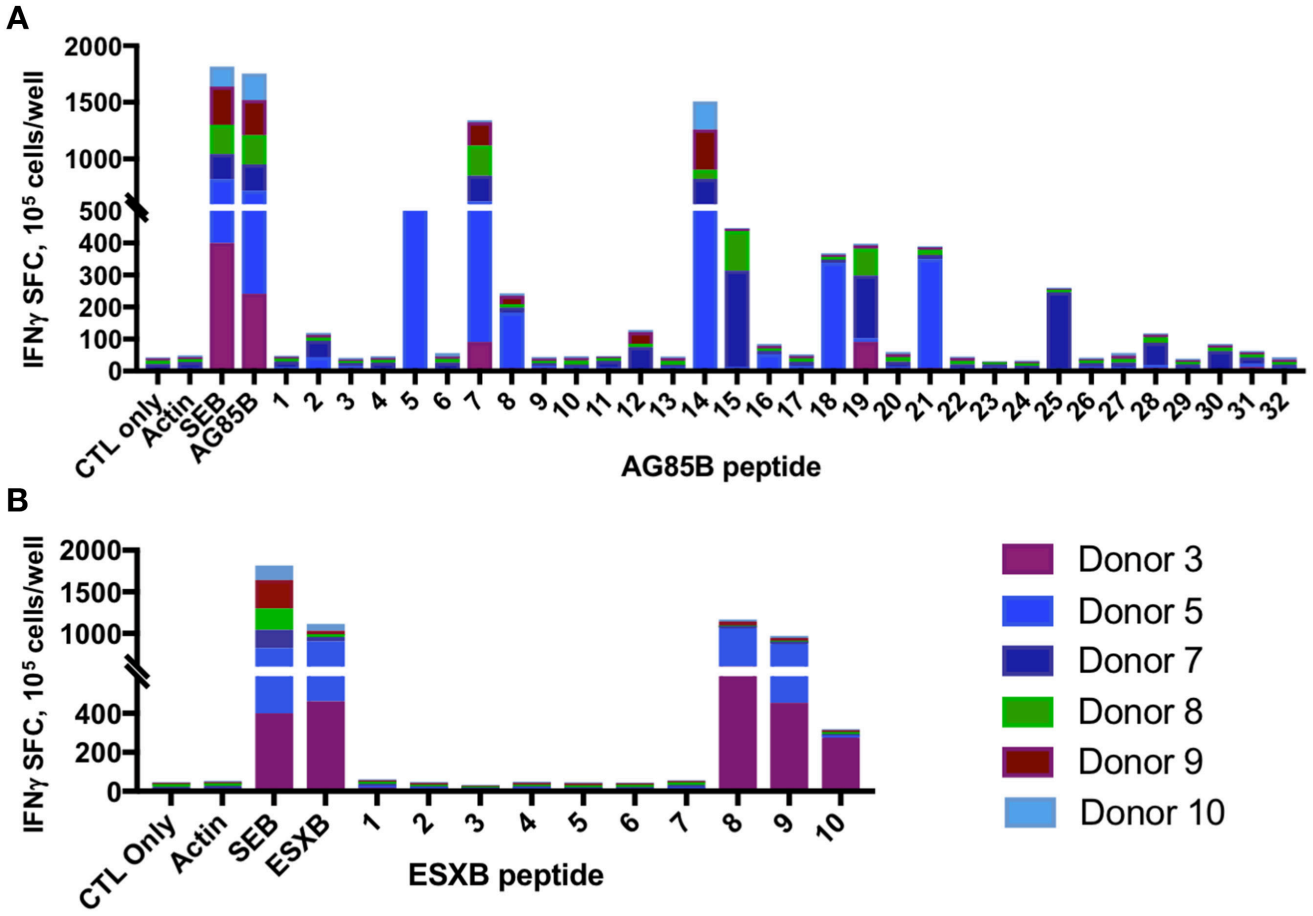
MSTs recognize multiple epitopes within AG85B and ESXB. Epitope mapping of AG85B **(A)** and ESXB **(B)** via IFN-γ ELISpot showed eight peptides from AG85B and three from ESXB recognized by MSTs from multiple healthy donors. SFC, spot forming colonies; SEB, staphylococcal enterotoxin B.

Mapping of epitope recognition within AG85B and ESXB utilizing IFN-γ ELISPOT demonstrated several epitopes within each antigen that were recognized by multiple donors. Within AG85B, five donors recognized peptides #7 and 14, encompassing amino acid positions 61–75 and 131–145 (Figure 7). Peptide 15 (AA 141–155) elicited a response in two donors, and Peptide 19 (AA 181–195) elicited a response in three donors. Within ESXB, peptides 8–10 at the C-terminus (AA 71–100) were recognized by three donors. Analysis of shared donor HLA alleles using predictive algorithms [NetMHC (http://www.cbs.dtu.dk/services/NetMHCII/), IEDB MHC Predictor (www.iedb.org)] (34, 35) suggested Class II MHC restrictions of the AG85B peptides through HLA DRB4 01:01, DPB1 04:01/02, DRB1 07:01, and DRB3 02:02, and Class II restrictions of ESXB peptides through HLA DQB1 03:01/02 and DRB4 01:01 (Table 1). Analysis of interspecies conservation of these epitopes showed a high degree of conservation of the AG85B epitopes (67–100%, Supplemental Figure 7), and low to moderate conservation of the ESXB epitopes (40–93%, Supplemental Figure 8).

**Table 1:** Predicted MHC Restrictions of T cell epitopes within AG85B and ESXB.

**Protein**	**Peptide**	**Donors**	**HLA-A**	**HLA-B**	**HLA-C**	**HLA- DQB1**	**HLA-DRB1**	**HLA-DRB3**	**HLA-DRB4**	**HLA-DPB1**	**IEDB prediction**	**NETMHC II prediction**
AG85B	DIKVQFQSGGNNSPA	Donor 3	02:01, 24:07	15:02, 35:05	04:01, 08:01	03:01, 03:03	09:01, 12:02	03:01	**01:01**	**04:02**, 05:01	DRB4 01:01 >> DRB1 07:01 > DRB3 02:02 > DPB1 04:01	DRB4 01:01
		Donor 7	03:01, 29:02	14:02, 35:01	04:01, 08:02	02:02, 05:03	14:54, **07:01**	**02:02**	**01:01**	**04:01**, 15:01		
		Donor 8	02:01, 24:02	14:01, 18:01	08:02, 12:03	02:02, 03:01	11:04, **07:01**	**02:02**	**01:01**	04:02, 06:01		
		Donor 9	11:01, 24:02	15:02, 48:02	04:01, 08:01	03:02, 05:01	10:01, 04:11		**01:03**	**04:02**, 10:01		
	GCQTYKWETFLTSEL	Donor 5	02:01, 24:07	15:02, 35:05	04:01, 08:01	03:01, 03:03	09:01, 12:02	03:01	01:01	04:02, 05:01	DPB1 04:01 > DRB4 01:01	**DPB1 04:02 >** **DPB1 04:01**
		Donor 7	03:01, 29:02	14:02, 35:01	04:01, 08:02	02:02, 05:03	14:54, 07:01	02:02	**01:01**	**04:01**, 15:01		
		Donor 9	11:01, 24:02	15:02, 48:02	04:01, 08:01	03:02, 05:01	10:01, 04:11		**01:03**	**04:02**, 10:01		
		Donor 10	01:xx, 24:xx	08:xx, 58:xx	03:xx, 07:xx	02:xx	03:xx	02:xx, 01:xx				
	LTSELPQWLSANRAV	Donor 7	03:01, 29:02	14:02, 35:01	04:01, 08:02	02:02, 05:03	14:54, **07:01**	**02:02**	**01:01**	04:01, 15:01	DRB1 07:01 > DRB4 01:01 > DRB3 02:02	DRB3 02:02 > DRB1 07:01
		Donor 8	02:01, 24:02	14:01, 18:01	08:02, 12:03	02:02, 03:01	11:04, **07:01**	**02:02**	**01:01**	04:02, 06:01		
	QQFIYAGSLSALLDP	Donor 3	02:01, 24:07	15:02, 35:05	04:01, 08:01	03:01, 03:03	09:01, 12:02	03:01	01:01	**04:02**, 05:01	DRB1 07:01 > DRB3 02:02 > DPB1 04:02	DRB1 07:01 > DRB3 02:02 > DPB1 04:02
		Donor 7	03:01, 29:02	14:02, 35:01	04:01, 08:02	02:02, 05:03	14:54, **07:01**	02:02	01:01	**04:01**, 15:01		
		Donor 8	02:01, 24:02	14:01, 18:01	08:02, 12:03	02:02, 03:01	11:04, **07:01**	02:02	01:01	**04:02**, 06:01		
ESXB	EISTNIRQAGVQYSR	Donor 3	02:01, 24:07	15:02, 35:05	04:01, 08:01	**03:01, 03:03**	09:01, 12:02	03:01	**01:01**	**04:02**, 05:01	DRB4 01:01 > DQB1 03:02	DQB1 03:01 > DRB4 01:01
		Donor 9	11:01, 24:02	15:02, 48:02	04:01, 08:01	**03:02**, 05:01	10:01, 04:11		**01:03**	**04:02**, 10:01		
	VQYSRADEEQQQALS	Donor 3	02:01, 24:07	15:02, 35:05	04:01, 08:01	**03:01, 03:03**	09:01, 12:02	03:01	**01:01**	**04:02**, 05:01	DQB1 03:02 > DRB4 01:01	DQB1 03:02
		Donor 9	11:01, 24:02	15:02, 48:02	04:01, 08:01	**03:02**, 05:01	10:01, 04:11		**01:03**	**04:02**, 10:01		

*Bold significant shared HLA alleles and Blue significant top predicted HLA restriction*.

## Discussion

Mycobacterial infections are common in immunocompromised hosts, and treatment can be exceedingly challenging. Even among immunocompetent individuals, multi-drug resistant tuberculosis is an emerging problem, with resistance to first line antimycobacterial agents reported in 4% of new cases and 21% of previously treated cases worldwide (1). In infants with SCID or similarly profound forms of PID, clearance of mycobacterial infections is often impossible without restoration of T cell immunity (2). The use of repeated whole blood transfusions from a BCG-immunized sibling was reported as adjunctive therapy for an infant with SCID with improvement in BCGosis (36). Accordingly, adoptive immunotherapy targeting mycobacteria could be a useful adjunctive therapy alongside antibiotics.

Our analysis of the functionality of MSTs derived from healthy donors demonstrated that responses to the selected mycobacterial antigens were CD4^+^ restricted and polyfunctional. All donors (BCG vaccinated or otherwise) recognized at least one antigen. Analysis of responses between BCG-vaccinated and unvaccinated showed a difference in response magnitude on ELISPOT for PPE68, but not for the other four antigens. ESXA and ESXB were recognized by both donor groups, in spite of the fact that these genes are deleted in BCG. None of the donors had prior histories of tuberculosis infection. This may suggest that the reactivity to EXSA and ESXB (as well as the other antigens in the non-vaccinated donors) represents prior responses to other encountered mycobacterial species. If true, this would support the existence of cross-reactive epitopes shared amongst these species. Multiplex cytokine analysis showed consistent IFN-γ, TNF, and GM-CSF production in response to antigen restimulation, as well as IL-13 and MIP1a in a subset of donors. GM-CSF production has been described in the setting of experimental mycobacterial infection, though its role in human infection is less clear (37). IL-13 is a Th2 cytokine associated with fibrosis and mucus production, and was only noted in BCG-unvaccinated donors. It is possible that BCG vaccination may be the cause of the absence of IL-13 in vaccinated donors, and may reinforce a Th1 skewed cytokine response to these antigens in vaccinated individuals. Many studies have highlighted the importance of Th1 CD4^+^ T cell responses in activating macrophages to control mycobacterial disease (38). In experimental models, Th2 cytokines have been associated with progression of mycobacterial infections, though in human tuberculosis, it is unclear if elevated Th2 cytokine profiles are a cause or consequence of mycobacterial infections.

In adoptive immunotherapy with partially HLA-matched virus-specific T cells, the HLA matching algorithm between the VST donor and recipient appears to be one of the key steps in improving the efficacy of this therapy, as identification of the HLA restriction of one or more immunodominant viral epitopes has correlated with antiviral activity *in vivo* (39). Mapping of mycobacterial epitopes and HLA restrictions would likely also be essential for “off the shelf” use of partially matched MSTs. Here, we describe several novel epitopes within AG85B and ESXB. Within AG85B, the recognized protein regions (AA 61–75, 131–145, 141–155, 180–195) were highly stable across species. Prior studies have shown that these regions are involved in secondary structure formation, which may explain their relative stability. Amino acids 181–195 overlapped with a domain in AG85B that was previously predicted to contain T cell epitopes and elicited *ex vivo* CD4^+^ T cell proliferation (31). Recognized epitopes within the C-terminus of ESXB were more variable across species. This region of the protein has been described to be essential for monocyte binding of the ESXB complex, and accordingly may play an important role in mycobacterial pathogenesis (40). It has been postulated that ESXA/ESXB deletion contributes to the attenuation of BCG. Further testing of additional donors with a wide breadth of HLA types would be needed to better understand the breadth of HLA restrictions of these antigens as well as the stability of epitopes in clinically isolated mycobacterial species. Comparison of published protein sequences across different mycobacterial species shows differing degrees of homology (Supplemental Table 8).

The genomes of mycobacterial species average 2 MB with >2,000 described genes in many species. Accordingly, there are likely a vast number of immunogenic proteins beyond the five antigens tested in this study. However, use of *M. tuberculosis* lysate and *M. avium* Sensitin as non-biased antigen sources still yielded reactivity to the selected proteins. Though the breadth of antigen responses is likely much broader than the selected proteins, they were not overshadowed due to antigenic competition during expansion. Previous studies have similarly described cross reactivity between *M. tuberculosis* and non-tuberculous mycobacteria, though the biologic importance of immunologic responses to these shared antigens remains unclear (41).

There is also evidence that γ/δ T-cells are activated by phosphate antigens from mycobacteria, though their role in the control of mycobacterial infections remains unclear (42). However, we did not observe expansion of γ/δ T-cells even when utilizing whole cell lysates from *M. tuberculosis*, which contains lipids and carbohydrates in addition to proteins.

Though T cell immunity is clearly important for anti-mycobacterial defense, myeloid cells are also essential, as demonstrated by many forms of primary immunodeficiency such as *GATA2* haploinsufficiency, *IFNGR1/2* deficiency, Chronic granulomatous disease, and *IRF8* deficiency. Of the tested patients with PID, responses to mycobacterial antigens were only found in two patients with *NFKB1* haploinsufficiency (two of five antigens) and NEMO (one of five antigens). Responses were detectable in both tested patients with anti-IFN-γ autoantibodies, which was expected with *ex vivo* expansion of these patient's cells in the absence of patient serum. *NFKB1* and related disorders have been well-described to cause impairment of T cell proliferation, and subtle T cell abnormalities have also been described in *IFNGR1* deficiency (43, 44). T cell lymphopenia has been also described in *GATA2* haploinsufficiency (45).

In this study, we have shown that mycobacterial-specific T cells can be reliably expanded from healthy donors using a rapid expansion protocol that is compatible with good manufacturing practices. Though T cell therapy alone would likely not be helpful for forms of PID with predominantly myeloid defects, one could envision usage of MSTs shortly after myeloid engraftment post-transplant in order to hasten recovery of T cell control of infection, which would otherwise not be expected to occur until months later. Though further work will be necessary to better characterize ideal donors, antigens, and T cell characteristics, T cell immunotherapy targeting mycobacteria could be a useful future treatment for patients with invasive mycobacterial infections.

## Data Availability

All datasets generated for this study are included in the manuscript and/or the Supplementary Files.

## Ethics Statement

This study was carried out in accordance with the recommendations of the Institutional Review Boards at the local institutions (Children's National Health System, the National Institute of Allergy and Infectious Diseases, and All Children's Hospital) with written informed consent from all subjects. All subjects gave written informed consent in accordance with the Declaration of Helsinki. The protocols were approved by each local Institutional Review Board (Children's National Health System, the National Institute of Allergy and Infectious Diseases, and All Children's Hospital).

## Author Contributions

SP, GS, CB, and MK designed and performed research, analyzed data, and wrote the paper. HL, MG, and YW designed and performed research and analyzed data. AF, JL, SH, and BO contributed patients, analyzed data, and reviewed the paper. PH and CC designed research, analyzed data, and wrote the paper. BO designed research and analyzed data. CP designed and performed research.

### Conflict of Interest Statement

CB is a founder of Mana Therapeutics and serves on the scientific advisory boards of Cellectis, Torque and NexImmune. PH is a founder of Mana Therapeutics. CC is a founder of Mana Therapeutics. The remaining authors declare that the research was conducted in the absence of any commercial or financial relationships that could be construed as a potential conflict of interest.

## Abbreviations

BCG: bacillus calmette guerin
CTL: cytotoxic T lymphocyte
G: gravity
HSCT: hematopoietic stem cell transplantation
IFN: interferon
IL: interleukin
MSMD: mendelian susceptibility to mycobacterial disease
MST: mycobacteria-specific T cells
PBMC: peripheral blood mononuclear cells
PID: primary immunodeficiency
SCID: severe combined immunodeficiency
VST: virus-specific T cells
SEB: staphylococcus enterotoxin B.
