# Supplementary figures and images for: Mycobacteria-Specific T Cells May Be Expanded From Healthy Donors and Are Near Absent in Primary Immunodeficiency Disorders
Source: Front Immunol. 2019 Mar 29;10:621. doi: 10.3389/fimmu.2019.00621 (PMC6450173; doi:10.3389/fimmu.2019.00621)

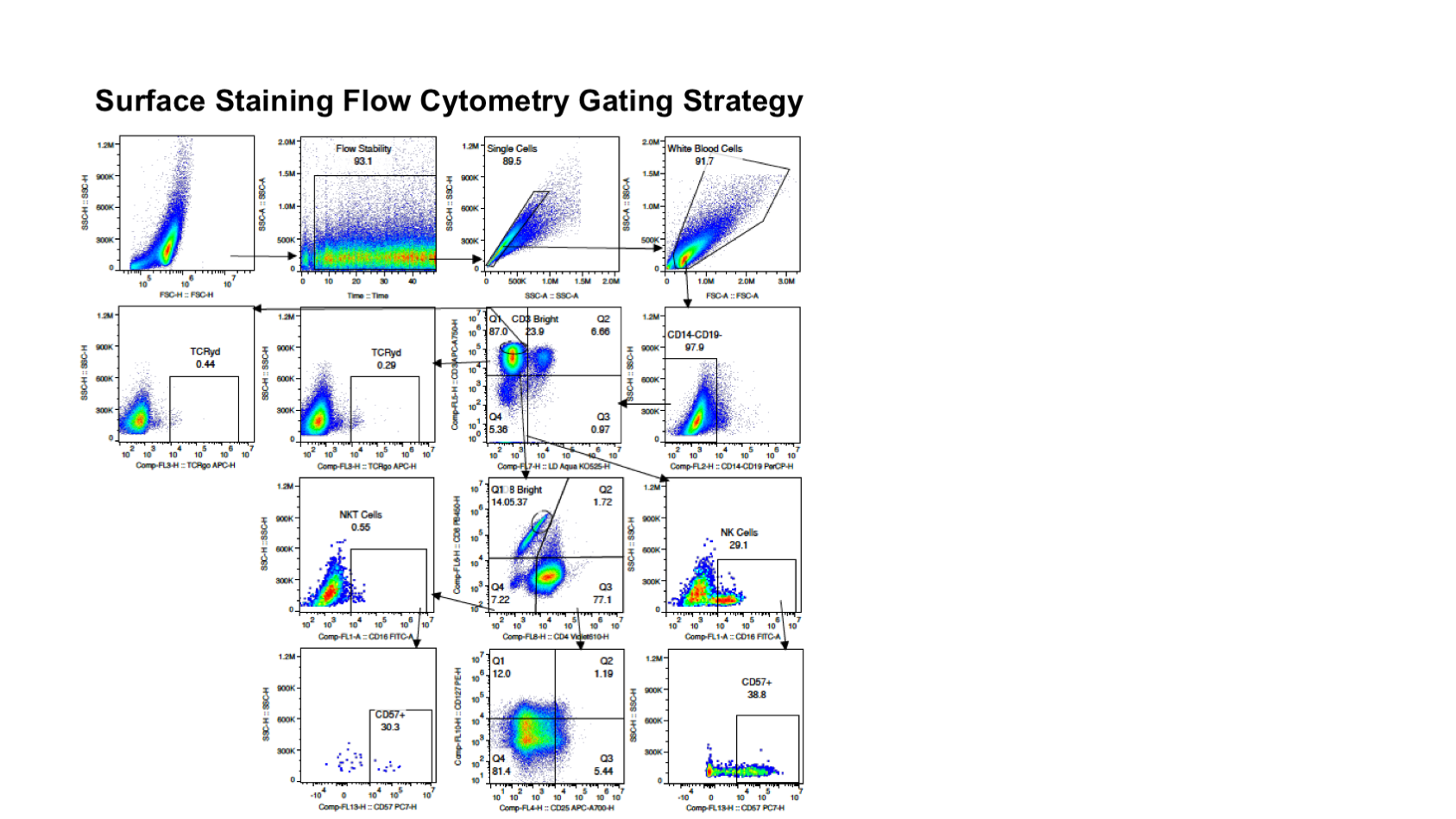

Supplement: Supplementary file 1 [file Image_1.TIFF]

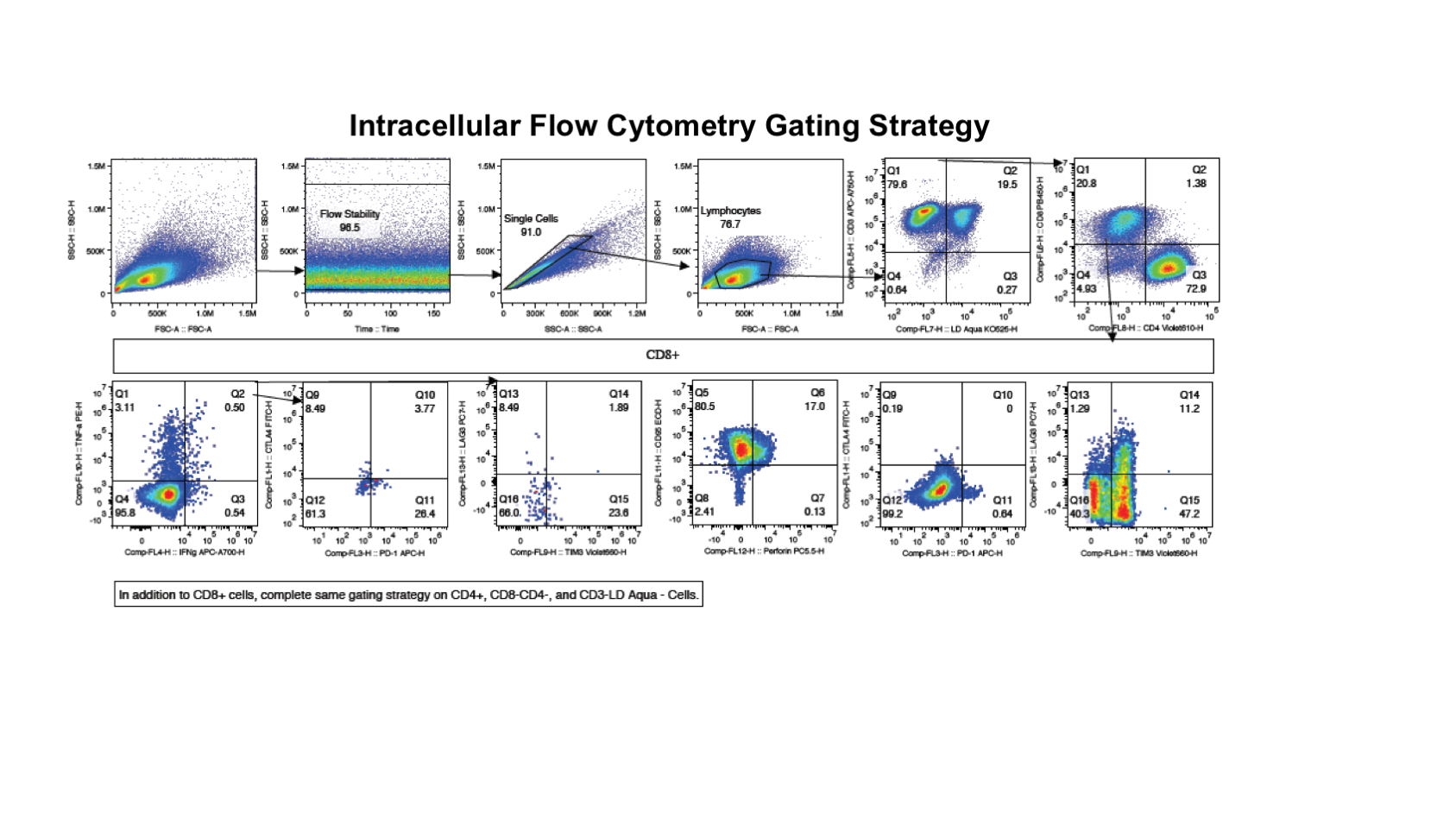

Supplement: Supplementary file 2 [file Image_2.TIFF]

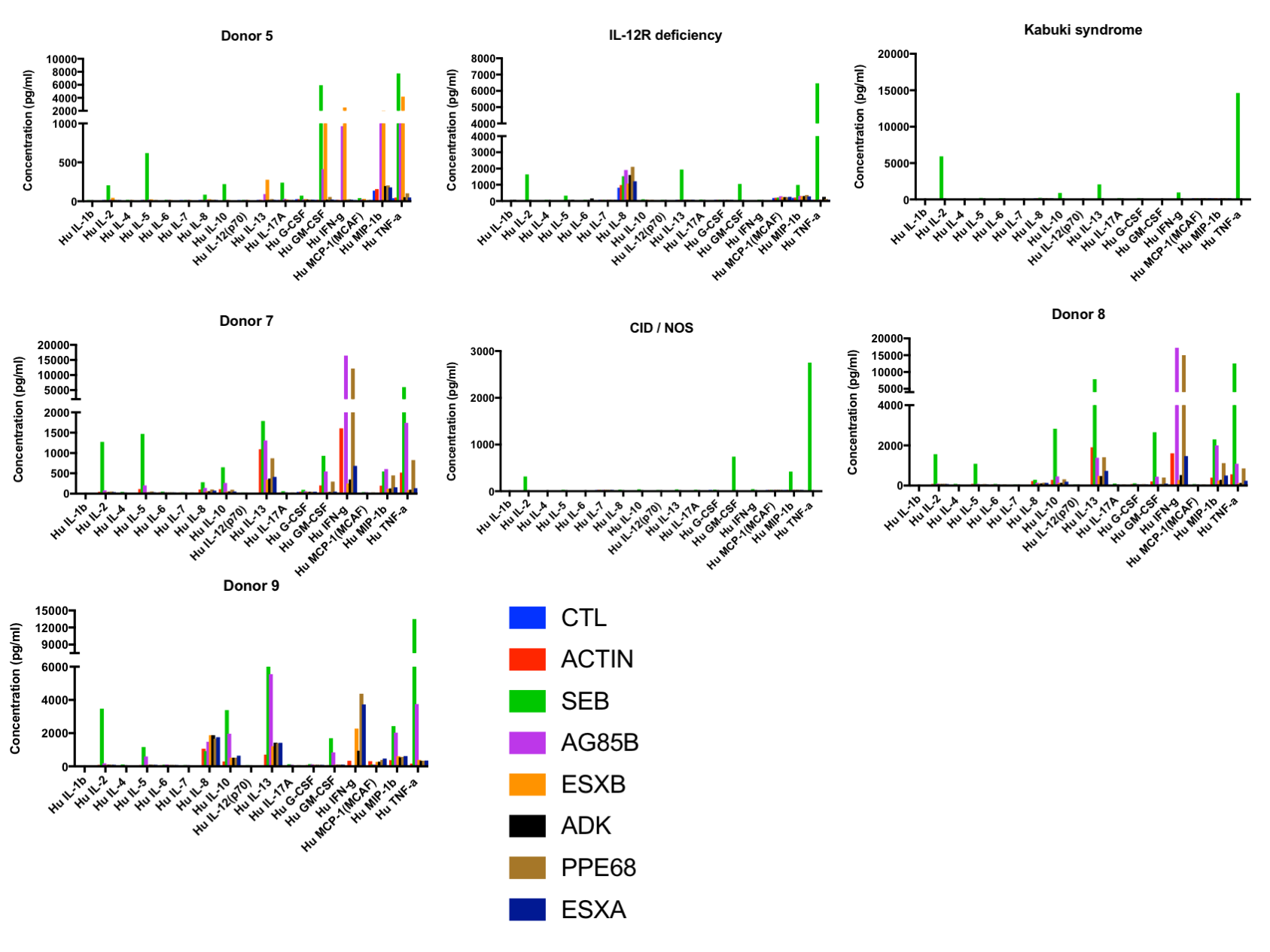

Supplement: Supplementary file 3 [file Image_3.TIFF]

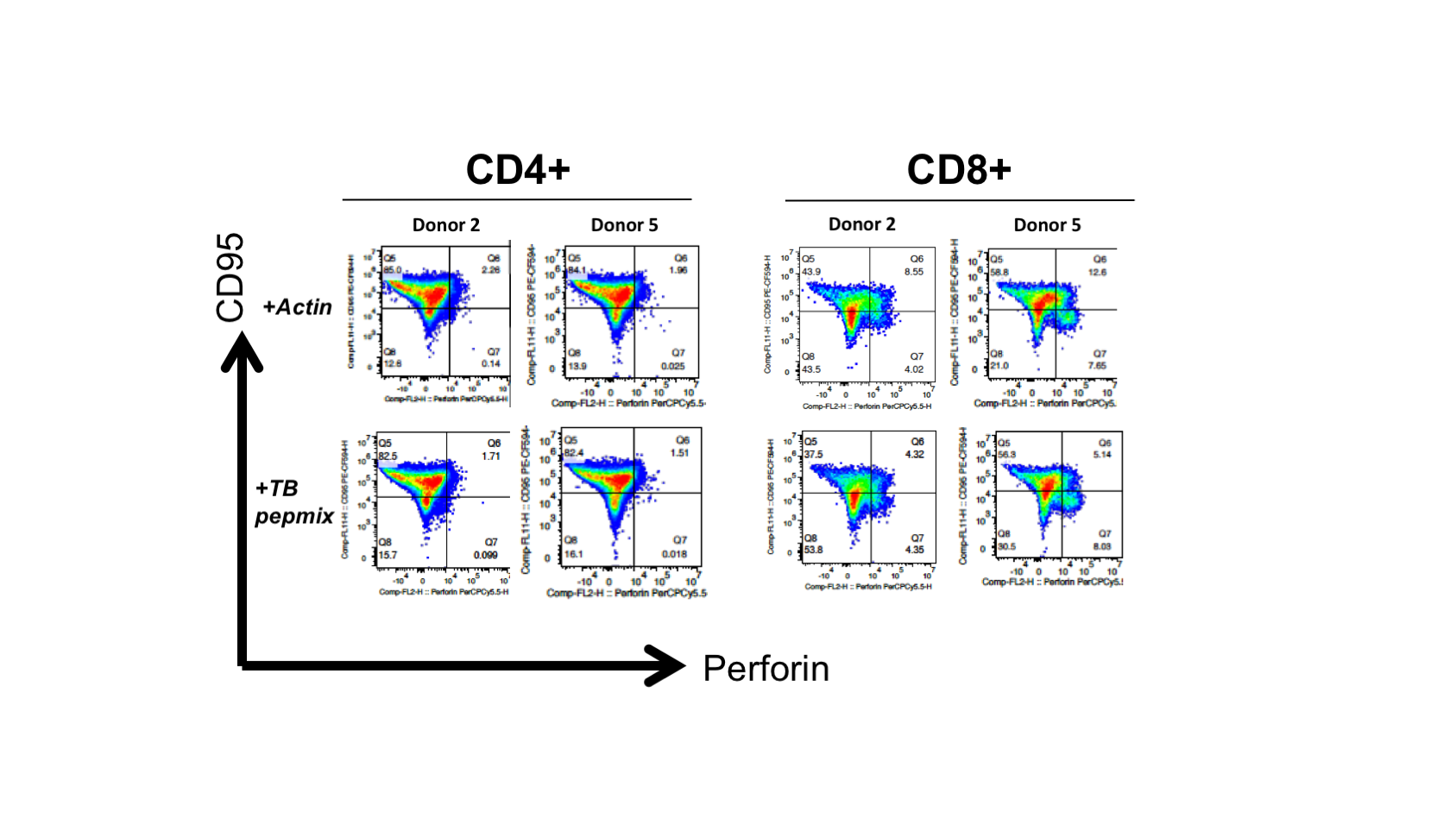

Supplement: Supplementary file 4 [file Image_4.TIFF]

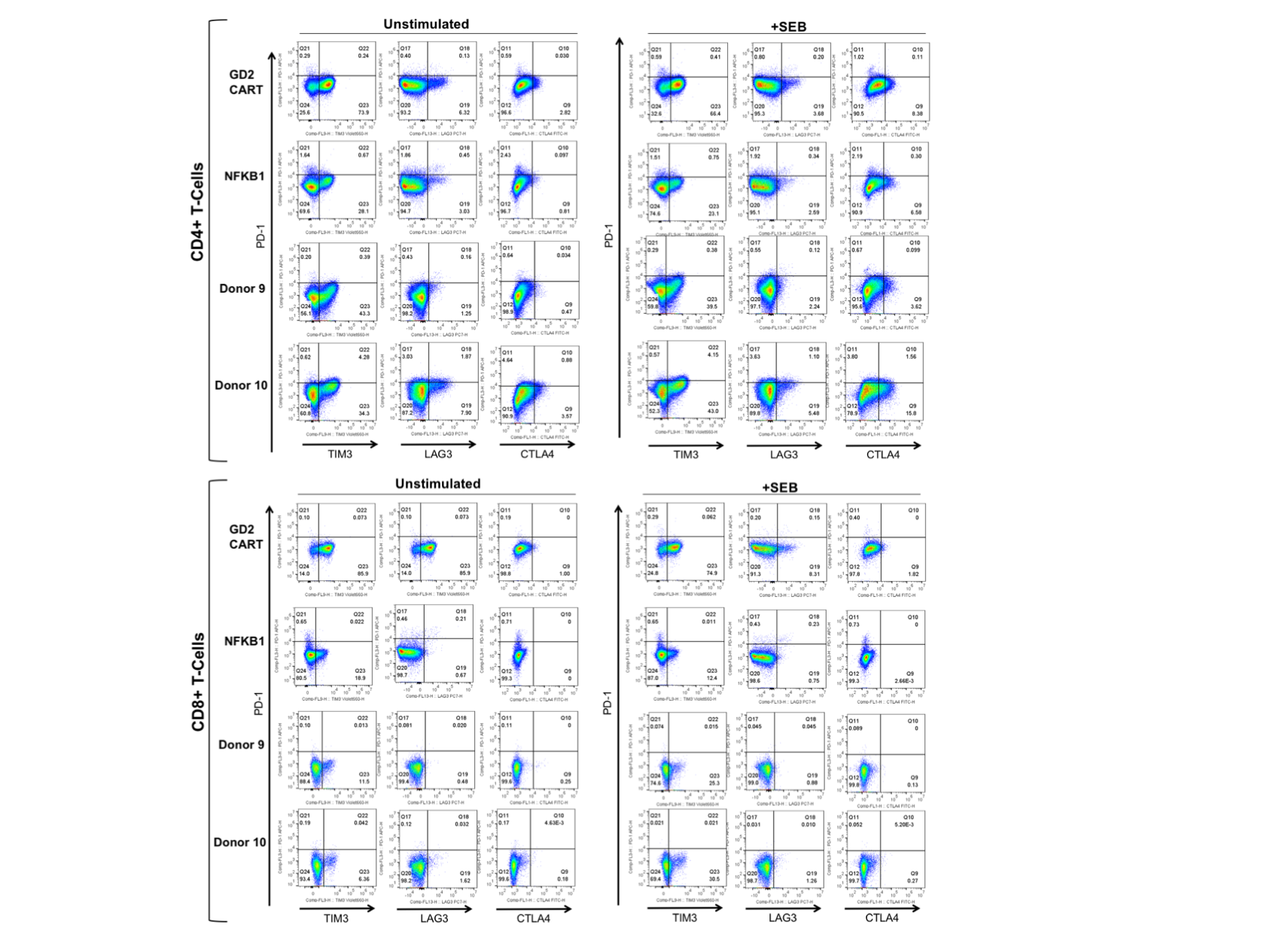

Supplement: Supplementary file 5 [file Image_5.TIFF]

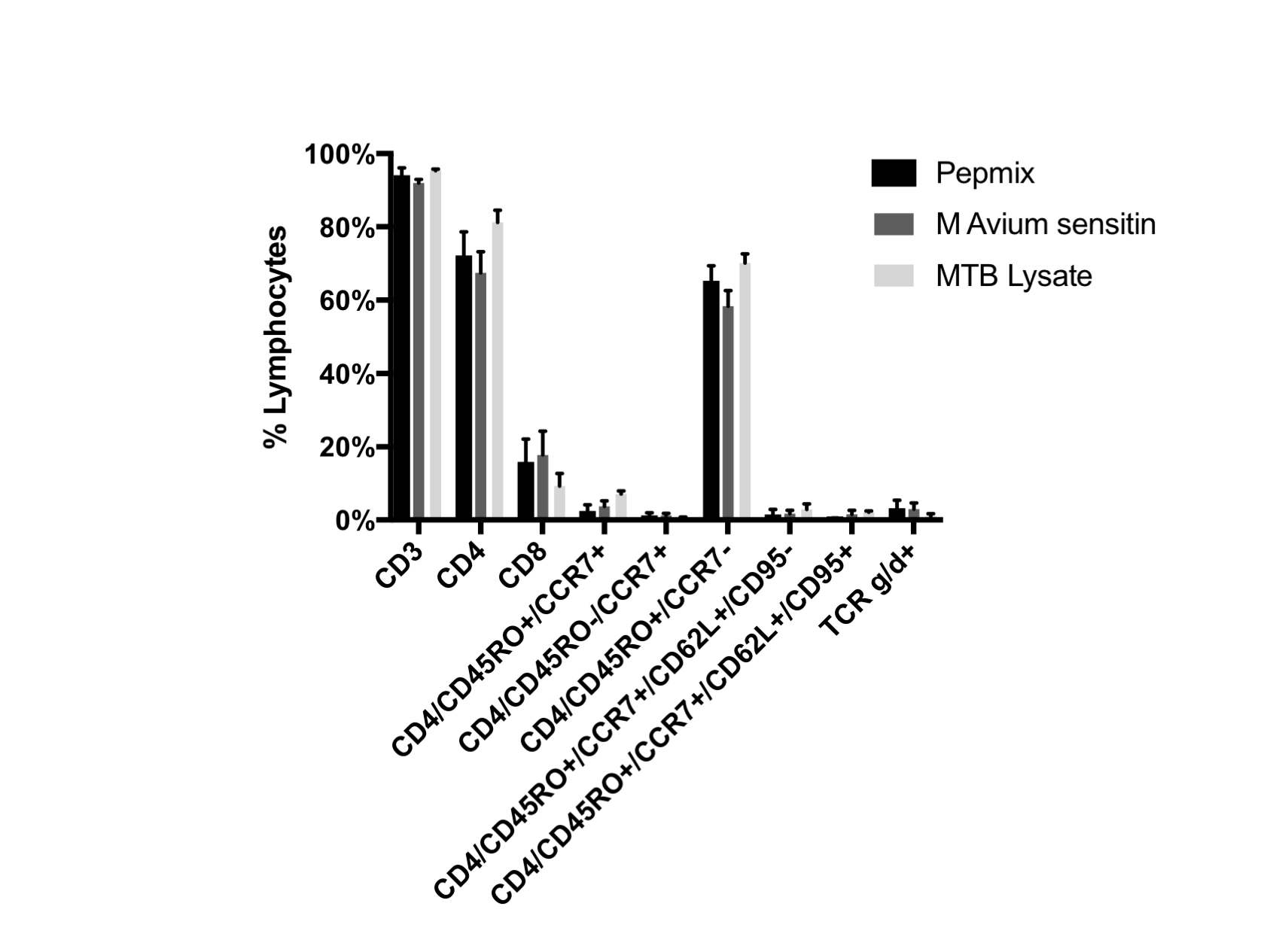

Supplement: Supplementary file 6 [file Image_6.TIFF]

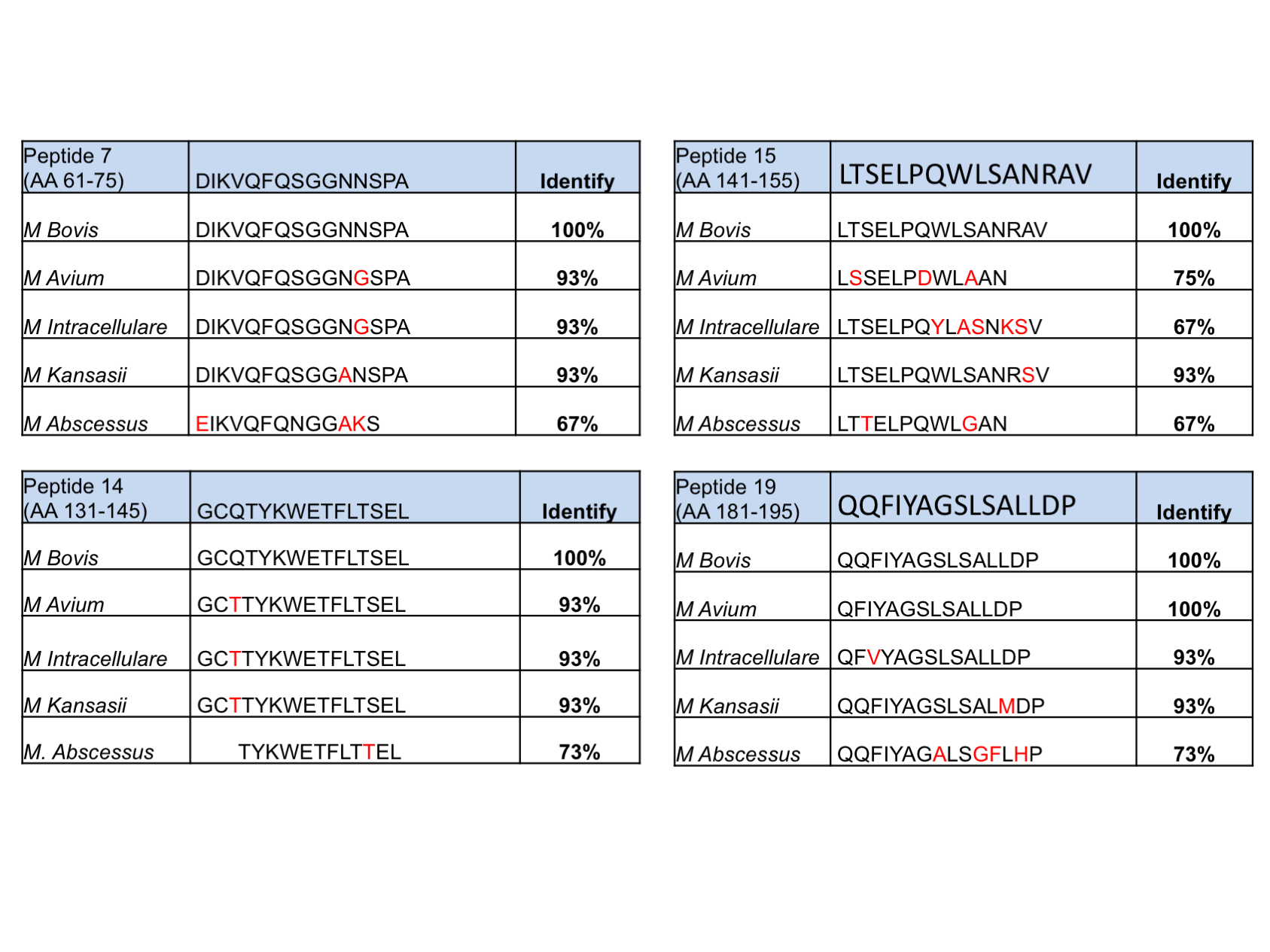

Supplement: Supplementary file 7 [file Image_7.TIFF]

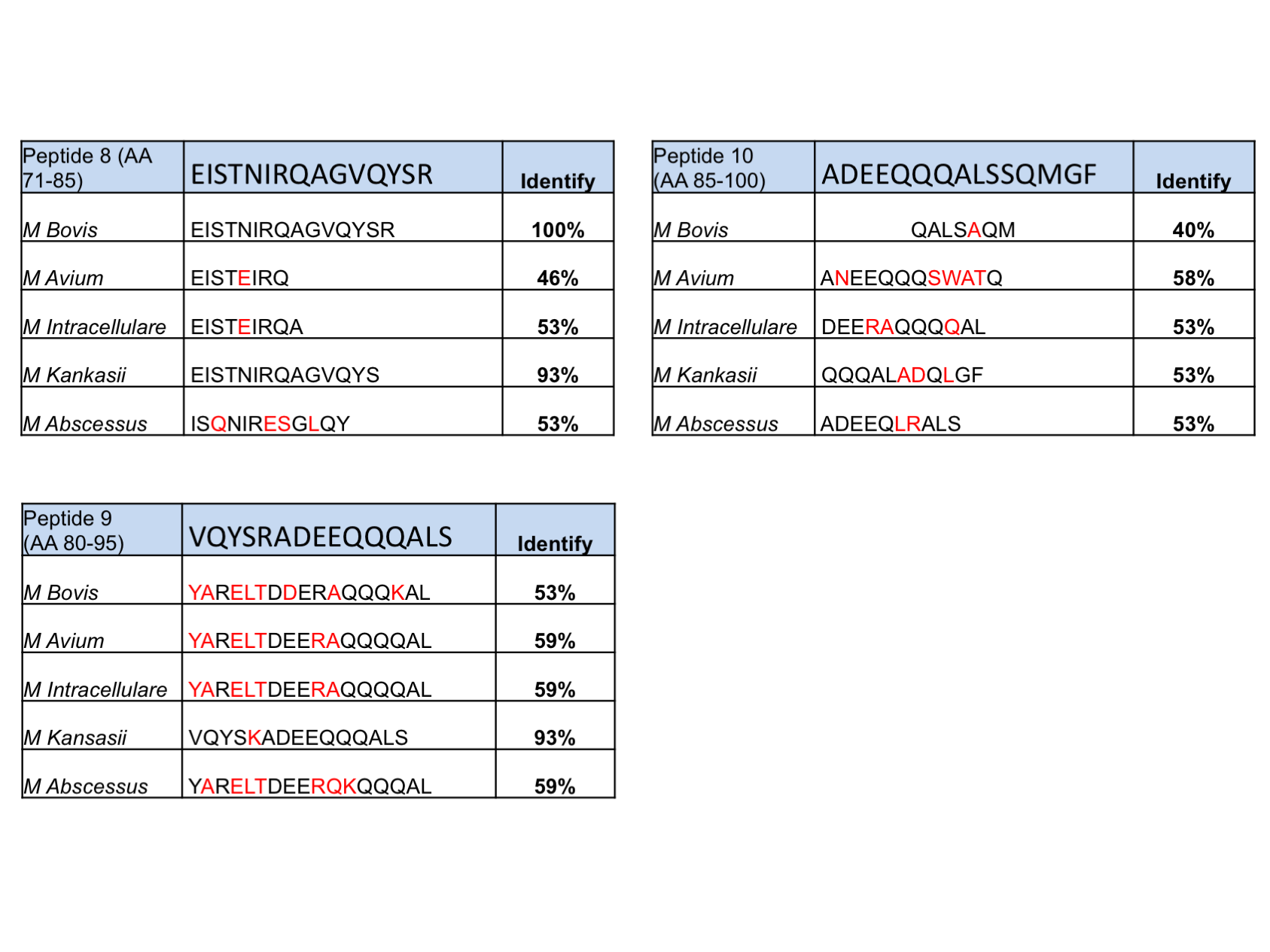

Supplement: Supplementary file 8 [file Image_8.TIFF]
