# Supplementary material for: Mycobacteria-Specific T Cells May Be Expanded From Healthy Donors and Are Near Absent in Primary Immunodeficiency Disorders
Source: Front Immunol. 2019 Mar 29;10:621. doi: 10.3389/fimmu.2019.00621 (PMC6450173; doi:10.3389/fimmu.2019.00621)
Supplement: Supplementary file 9 [file Table_1.DOCX]

**Supplemental Table 1:** Diagnoses of Primary Immunodeficiency Patients and Infection Details

| **Subject #** | **Disorder** | **Mycobacterial species** | **Sites of infection** |
| --- | --- | --- | --- |
| 1 | CID/NOS | *M. avium* complex | Mediastinal lymphadenitis |
| 2 | Kabuki syndrome | *M. abscessus* | Pulmonary infection |
| 3 | IFNGR1 deficiency | *M. avium* | Bacteremia, GI tract, mesenteric nodes |
| 4 | GATA2 haploinsufficiency | *M. avium* | Pulmonary infection |
| 5 | IL12RB1 deficiency | *M. avium* | Bacteremia, GI tract, pulmonary infection |
| 6 | NEMO | *M. avium* | Bacteremia, colitis |
| 7 | IFN-γ Autoantibody | *M. abscessus* | Blood, soft tissue, bone |
| 8 | IFN-γ Autoantibody | *M. avium* complex | Soft tissue, bone |

**Supplemental Table 2**: Mycobacterial Antigen Refseq numbers

| **Antigen** | **RefSeq** |
| --- | --- |
| AG85B | NP_216402.1 |
| PPE68 | WP_003399879.1 |
| ESXA | WP_00033999963.1 |
| ESXB | NP_218391.1 |
| ADK | NP_215247.1 |

**Supplemental Table 3: Conjugated Antibody Fluorophores and Clones**

| **Company** | **Marker** | **Fluorophore** | **Clone** |
| --- | --- | --- | --- |
| BioLegend | CD16 | FITC | 3G8 |
| BioLegend | CD127 | PE | A019D5 |
| Miltenyi | CD14 | PerCP | TUK4 |
| BioLegend | CD19 | PerCP Cy5.5 | HIB19 |
| MIltenyi | CD57 | PE-Vio770 | REA769 |
| BioLegend | TCRgd | APC | B1 |
| BioLegend | CD25 | Ax700 | BC96 |
| BioLegend | CD3 | APC-Fire750 | SK7 |
| BioLegend | CD8 | BV421 | RPA-T8 |
| BioLegend | CD4 | BV605 | DREG-56 |
| BioLegend | CD27 | FITC | O323 |
| BioLegend | CD95 | PE-Daz594 | OKT4 |
| BD | CD28 | PECy5 | CD28.2 (RUO) |
| Miltenyi | CD45RO | PE-Vio770 | REA611 |
| BioLegend | CCR7 | Ax700 | G043H7 |
| BioLegend | CD62L | BV650 | DREG-56 |
| eBioscience | CTLA4 | FITC | 14D3 |
| BioLegend | TNFa | PE | MAb11 |
| BioLegend | Perforin | PerCP Cy5.5 | B-D48 |
| BioLegend | CD223 (LAG3) | PE-Cy7 | 11C3C65 |
| BD | PD-1 | APC | MIH4 |
| Invitrogen | IFNy | Ax700 | 4S.B3 |
| BioLegend | CD366 (TIM3) | BV650 | F38-2E2 |

**Supplemental Table 4:** Statistical Analysis of Donor Antigen Specificity

| **Donor** | **Condition** | **IFN-γ SPW** | | | **Mean** | **P value**  **(vs Actin)** |
| --- | --- | --- | --- | --- | --- | --- |
| Donor 1 | CTL only | 5 | 0 | 1 | 2.00 |  |
|  | Actin | 1 | 2 | 2 | 1.67 |  |
|  | SEB | 483 | 455 | 456 | 464.67 |  |
|  | AG85A | 11 | 14 | 17 | 14.00 | **0.013596339** |
|  | PPE68 | 6 | 2 | 2 | 3.33 | 0.422649731 |
|  | ESAT6 | 0 | 1 | 27 | 9.33 | 0.469685578 |
|  | ESXB | 53 | 48 | 42 | 47.67 | **0.005623287** |
|  | ADK | 1 | 0 | 0 | 0.33 | 0.183503419 |
| Donor 2 | CTL only | 1 | 2 | 1 | 1.33 |  |
|  | Actin | 4 | 0 | 4 | 2.67 |  |
|  | SEB | 408 | 409 | 444 | 420.33 |  |
|  | AG85A | 114 | 173 | 133 | 140.00 | **0.017961432** |
|  | PPE68 | 46 | 33 | 28 | 35.67 | **0.02390784** |
|  | ESAT6 | 23 | 32 | 34 | 29.67 | **0.021679195** |
|  | ESXB | 25 | 29 | 28 | 27.33 | **0.008829797** |
|  | ADK | 0 | 0 | 0 | 0.00 | 0.183503419 |
| Donor 3 | CTL only | 0 | 0 | 0 | 0.00 |  |
|  | Actin | 0 | 1 | 1 | 0.67 |  |
|  | SEB | 517 | 500 | 523 | 513.33 |  |
|  | AG85A | 51 | 49 | 62 | 54.00 | **0.005385862** |
|  | PPE68 | 1 | 2 | 2 | 1.67 | 0.101191507 |
|  | ESAT6 | 12 | 11 | 14 | 12.33 | **0.005665768** |
|  | ESXB | 608 | 584 | 601 | 597.67 | **0.000152412** |
|  | ADK | 0 | 0 | 1 | 0.33 | 0.422649731 |
| Donor 4 | CTL only | 1 | 0 | 1 | 0.67 |  |
|  | Actin | 0 | 2 | 0 | 0.67 |  |
|  | SEB | 452 | 358 | 381 | 397.00 |  |
|  | AG85A | 18 | 29 | 18 | 21.67 | **0.019803941** |
|  | PPE68 | 2 | 5 | 6 | 4.33 | 0.092735291 |
|  | ESAT6 | 0 | 1 | 0 | 0.33 | 0.422649731 |
|  | ESXB | 1 | 1 | 0 | 0.67 | **1** |
|  | ADK | 1 | 0 | 0 | 0.33 | 0.74180111 |
| Donor 5 | CTL only | 6 | 0 | 1 | 2.33 |  |
|  | Actin | 7 | 7 | 4 | 6.00 |  |
|  | SEB | 596 | 585 | 519 | 566.67 |  |
|  | AG85A | 501 | 581 | 529 | 537.00 | **0.001917902** |
|  | PPE68 | 107 | 80 | 102 | 96.33 | **0.009119242** |
|  | ESAT6 | 29 | 29 | 38 | 32.00 | **0.022860164** |
|  | ESXB | 569 | 428 | 547 | 514.67 | **0.007457789** |
|  | ADK | 13 | 10 | 9 | 10.67 | **0.033908217** |
| Donor 6 | CTL only | 0 | 1 | 1 | 0.67 |  |
|  | Actin | 0 | 1 | 0 | 0.33 |  |
|  | SEB | 478 | 497 | 456 | 477.00 |  |
|  | AG85A | 7 | 6 | 5 | 6.00 | **0.01355995** |
|  | PPE68 | 15 | 29 | 19 | 21.00 | **0.032901347** |
|  | ESAT6 | 1 | 0 | 0 | 0.33 | 1 |
|  | ESXB | 1 | 0 | 0 | 0.33 | **1** |
|  | ADK | 0 | 1 | 2 | 1.00 | 0.422649731 |
| Donor 7 | CTL alone | 8 | 5 |  | 6.50 |  |
|  | Actin | 2 | 5 |  | 3.50 |  |
|  | SEB | 604 | 576 |  | 590.00 |  |
|  | AG85A | 385 | 400 |  | 392.50 | **0.000386371** |
|  | PPE68 | 386 | 374 |  | 380.00 | **0.000269728** |
|  | ESAT6 | 95 | 104 |  | 99.50 | **0.002432502** |
|  | ESXB | 22 | 30 |  | 26.00 | **0.034210227** |
|  | ADK | 31 | 32 |  | 31.50 | **0.003173604** |
| Donor 8 | CTL alone | 0 | 1 |  | 0.50 |  |
|  | Actin | 0 | 0 |  | 0.00 |  |
|  | SEB | 561 | 895 |  | 728.00 |  |
|  | AG85A | 269 | 227 |  | 248.00 | **0.007094056** |
|  | PPE68 | 174 | 190 |  | 182.00 | **0.001926552** |
|  | ESAT6 | 64 | 75 |  | 69.50 | **0.006204393** |
|  | ESXB | 17 | 24 |  | 20.50 | **0.02793371** |
|  | ADK | 35 | 36 |  | 35.50 | **0.000198314** |
| Donor 9 | CTL alone | 3 | 2 |  | 2.50 |  |
|  | Actin | 9 | 6 |  | 7.50 |  |
|  | SEB | 1957 | 1584 |  | 1770.50 |  |
|  | AG85A | 466 | 419 |  | 442.50 | **0.002917556** |
|  | PPE68 | 135 | 127 |  | 131.00 | **0.001194401** |
|  | ESAT6 | 97 | 71 |  | 84.00 | **0.028037425** |
|  | ESXB | 37 | 47 |  | 42.00 | **0.022136969** |
|  | ADK | 27 | 35 |  | 31.00 | **0.031493807** |
| Donor 10 | CTL alone | 1 | 0 |  | 0.50 |  |
|  | Actin | 1 | 0 |  | 0.50 |  |
|  | SEB | 906 | 810 |  | 858.00 |  |
|  | AG85A | 111 | 109 |  | 110.00 | **0.000104235** |
|  | PPE68 | 318 | 283 |  | 300.50 | **0.003388257** |
|  | ESAT6 | 4 | 1 |  | 2.50 | 0.333333333 |
|  | ESXB | 6 | 7 |  | 6.50 | **0.013606076** |
|  | ADK | 1099 | 1042 |  | 1070.50 | **0.000708914** |

*(****bold****: meets criteria for antigen specificity)*

**Supplemental Table 5**: Statistical Analysis of MST Antigen Specificity of BCG Vaccinated versus Naïve Healthy Donors

| **Covariate** | **Statistics** | **BCG**  **Vaccinated** | **BCG Naive** | **Non-Parametric P-value** |
| --- | --- | --- | --- | --- |
| CTL_only | N | 5 | 5 | 0.834 |
|  | Mean | 1.27 | 2.13 |  |
|  | Median | 1.33 | 0.67 |  |
|  | Min | 0 | 0.5 |  |
|  | Max | 2.33 | 6.5 |  |
|  | Std Dev | 0.95 | 2.58 |  |
| Actin | N | 5 | 5 | 0.463 |
|  | Mean | 2.34 | 2.37 |  |
|  | Median | 1.67 | 0.5 |  |
|  | Min | 0.67 | 0 |  |
|  | Max | 6 | 7.5 |  |
|  | Std Dev | 2.21 | 3.2 |  |
| AG85B | N | 5 | 5 | 0.602 |
|  | Mean | 153.33 | 239.8 |  |
|  | Median | 54 | 248 |  |
|  | Min | 14 | 6 |  |
|  | Max | 537 | 442.5 |  |
|  | Std Dev | 220.22 | 184.38 |  |
| PPE68 | N | 5 | 5 | **0.028** |
|  | Mean | 28.27 | 202.9 |  |
|  | Median | 4.33 | 182 |  |
|  | Min | 1.67 | 21 |  |
|  | Max | 96.33 | 380 |  |
|  | Std Dev | 40.59 | 141.05 |  |
| ESXA | N | 5 | 5 | 0.402 |
|  | Mean | 16.73 | 51.17 |  |
|  | Median | 12.33 | 69.5 |  |
|  | Min | 0.33 | 0.33 |  |
|  | Max | 32 | 99.5 |  |
|  | Std Dev | 13.64 | 46.65 |  |
| ESXB | N | 5 | 5 | 0.117 |
|  | Mean | 237.6 | 19.07 |  |
|  | Median | 47.67 | 20.5 |  |
|  | Min | 0.67 | 0.33 |  |
|  | Max | 597.67 | 42 |  |
|  | Std Dev | 292.76 | 16.47 |  |
| ADK | N | 5 | 5 | **0.015** |
|  | Mean | 2.33 | 233.9 |  |
|  | Median | 0.33 | 31.5 |  |
|  | Min | 0 | 1 |  |
|  | Max | 10.67 | 1070.5 |  |
|  | Std Dev | 4.66 | 467.88 |  |

**Supplemental Table 6:** Statistical Analysis of PID Patient Antigen Specificity

| **Donor** | **Condition** | **IFN-γ SPW** | | | **Mean** | **P value**  **(vs Actin)** |
| --- | --- | --- | --- | --- | --- | --- |
| IL12RB1 | CTL only | 0 | 0 |  | 0.00 |  |
|  | Actin | 2 | 1 |  | 1.50 |  |
|  | SEB | 324 | 354 |  | 339.00 |  |
|  | AG85A | 0 | 0 |  | 0.00 | 0.095465966 |
|  | PPE68 | 0 | 0 |  | 0.00 | 0.095465966 |
|  | ESAT6 | 0 | 0 |  | 0.00 | 0.095465966 |
|  | ESXB | 0 | 1 |  | 0.50 | 0.292893219 |
|  | ADK | 1 | 0 |  | 0.50 | 0.292893219 |
| Kabuki | CTL alone | 0 | 1 | 1 | 0.7 |  |
|  | Actin | 16 | 11 | 9 | 12.0 |  |
|  | SEB | 0 | 0 | 0 | 0.0 |  |
|  | AG85B | 0 | 0 | 2 | 0.7 | 0.048927794 |
|  | PPE68 | 0 | 3 | 1 | 1.3 | 0.057190958 |
|  | ESAT6 | 2 | 0 | 1 | 1.0 | 0.02390784 |
|  | ESXB | 2 | 2 | 2 | 2.0 | 0.040705781 |
|  | ADK | 3 | 0 | 5 | 2.7 | 0.075862293 |
| IFNGR1 | CTL alone | 3 | 1 | 3 | 2.3 |  |
|  | Actin | 3 | 0 | 1 | 1.3 |  |
|  | SEB | 442 | 495 | 377 | 438.0 |  |
|  | AG85B | 12 | 6 | 5 | 7.7 | 0.048810269 |
|  | PPE68 | 14 | 9 | 22 | 15.0 | 0.066492647 |
|  | ESAT6 | 2 | 4 | 3 | 3.0 | 0.370059212 |
|  | ESXB | 3 | 1 | 3 | 2.3 | 0.225403331 |
|  | ADK | 2 | 4 | 3 | 3.0 | 0.37005 |
| NEMO | Medium | 4 | 3 | 8 | 5.00 |  |
|  | Actin | 7 | 10 | 8 | 8.33 |  |
|  | SEB | 1068 | 1082.66667 | 1092 | 1080.89 |  |
|  | AG85B | 31 | 55 | 55 | 47.00 | **0.034338707** |
|  | PPE68 | 18 | 9 | 21 | 16.00 | 0.22156794 |
|  | ESAT6 | 8 | 10 | 20 | 12.67 | 0.376697708 |
|  | ESXB | 10 | 22 | 14 | 15.33 | 0.118082896 |
|  | ADK | 16 | 16 | 13 | 15.00 | 0.030996834 |
| NFKB1 | CTL only | 6 | 9 |  | 7.5 |  |
|  | Actin | 20 | 27 |  | 23.5 |  |
|  | SEB | 456 | 428 |  | 442.0 |  |
|  | AG85A | 396 | 381 |  | 388.5 | **0.000513772** |
|  | PPE68 | 41 | 39 |  | 40.0 | **0.045381191** |
|  | ESAT6 | 14 | 12 |  | 13.0 | 0.102104838 |
|  | ESXB | 7 | 14 |  | 10.5 | 0.1195289 |
|  | ADK | 10 | 9 |  | 9.5 | 0.058258088 |
| GATA2 | CTL only | 0 | 0 |  | 0.0 |  |
|  | Actin | 0 | 0 |  | 0.0 |  |
|  | SEB | 42 | 21 |  | 31.5 |  |
|  | AG85B | 0 | 0 |  | 0.0 | ND |
|  | PPE68 | 0 | 0 |  | 0.0 | ND |
|  | ESAT6 | 0 | 0 |  | 0.0 | ND |
|  | ESXB | 0 | 0 |  | 0.0 | ND |
|  | ADK | 0 | 0 |  | 0.0 | ND |
| CID | CTL only | 0 | 0 | 0 | 0.00 |  |
|  | Actin | 0 | 0 | 0 | 0.00 |  |
|  | SEB | 366 | 450 | 356 | 390.67 |  |
|  | AG85B | 1 | 0 | 1 | 0.67 | ND |
|  | PPE68 | 0 | 0 | 0 | 0.00 | ND |
|  | ESAT6 | 0 | 0 | 0 | 0.00 | ND |
|  | ESXB | 0 | 1 | 1 | 0.67 | ND |
|  | ADK | 1 | 0 | 0 | 0.33 | ND |
| IFNg AutoAb | CTL only | 3 | 2 |  | 2.5 |  |
|  | Actin | 2 | 3 |  | 2.5 |  |
|  | SEB | 973.142857 | 1157 |  | 1065.07143 |  |
|  | AG85B | 142 | 128 |  | 135 | **0.002793519** |
|  | PPE68 | 3 | 4 |  | 3.5 | 0.292893219 |
|  | ESAT6 | 16 | 23 |  | 19.5 | 0.040634498 |
|  | ESXB | 6 | 3 |  | 4.5 | 0.333333333 |
|  | ADK | 23 | 33 |  | 28 | **0.036706506** |
| IFNg AutoAb | CTL only | 20 | 2 |  | 11.00 |  |
|  | Actin | 2 | 2 |  | 2.00 |  |
|  | SEB | 1061.05263 | 1103.05263 |  | 1082.05 |  |
|  | AG85B | 12 | 22 |  | 17.00 | 0.095465966 |
|  | PPE68 | 2 | 4 |  | 3.00 | 0.422649731 |
|  | ESAT6 | 26 | 22 |  | 24.00 | **0.008163402** |
|  | ESXB | 34 | 38 |  | 36.00 | **0.003442351** |
|  | ADK | 16 | 16 |  | 16.00 | ND |

*(****bold****: meets criteria for antigen specificity)*

**Supplemental Table 7**: Statistical Analysis of MST Antigen Specificity by Culture Condition

|  | | | **Condition** | | | | | | | |  | |  |
| --- | --- | --- | --- | --- | --- | --- | --- | --- | --- | --- | --- | --- | --- |
|  | | | **___________________________** | | | | | | | |  | |  |
| **Covariate** | **Statistics** |  | **Pepmix N=5** | | **Sensitin N=5** | | | **Lysate N=5** | **Non-Parametric**  **P-value** | | |  |  |
| CTLonly | N |  | | 5 | | | 5 | 5 | 0.378 | | |  |  |
|  | Mean |  | | 2.13 | | | 6.93 | 4.87 |  |  |  |  |  |
|  | Median |  | | 0.67 | | | 7.33 | 4 |  |  |  |  |  |
|  | Min |  | | 0.5 | | | 0.33 | 1.67 |  |  |  |  |  |
|  | Max |  | | 6.5 | | | 13.67 | 8 |  |  |  |  |  |
|  | Std Dev |  | | 2.58 | | | 6.51 | 2.83 |  |  |  |  |  |
| Actin | N |  | 5 | | | 5 | | 5 | | 0.504 | |  | |
|  | Mean |  | 2.37 | | | 3.33 | | 4.67 | |  |  |  | |
|  | Median |  | 0.5 | | | 3.33 | | 2.33 | |  |  |  | |
|  | Min |  | 0 | | | 0 | | 1 | |  |  |  | |
|  | Max |  | 7.5 | | | 8.67 | | 13 | |  |  |  | |
|  | Std Dev |  | 3.2 | | | 3.43 | | 4.84 | |  |  |  | |
| AG85B | N |  | 5 | | | 5 | | 5 | | 0.221 | |  | |
|  | Mean |  | 239.8 | | | 88.93 | | 71.66 | |  |  |  | |
|  | Median |  | 248 | | | 97.67 | | 25.33 | |  |  |  | |
|  | Min |  | 6 | | | 0 | | 4 | |  |  |  | |
|  | Max |  | 442.5 | | | 169 | | 236.33 | |  |  |  | |
|  | Std Dev |  | 184.38 | | | 79.14 | | 96.92 | |  |  |  | |
| PPE68 | N |  | 5 | | | 5 | | 5 | | **0.032** | |  | |
|  | Mean |  | 202.9 | | | 15.6 | | 78.6 | |  |  |  | |
|  | Median |  | 182 | | | 15.67 | | 26.33 | |  |  |  | |
|  | Min |  | 21 | | | 1 | | 6.67 | |  |  |  | |
|  | Max |  | 380 | | | 37.67 | | 271.67 | |  |  |  | |
|  | Std Dev |  | 141.05 | | | 14.11 | | 109.76 | |  |  |  | |
| ESXA | N |  | 5 | | | 5 | | 5 | | 0.431 | |  | |
|  | Mean |  | 51.17 | | | 9 | | 10.07 | |  |  |  | |
|  | Median |  | 69.5 | | | 2 | | 5 | |  |  |  | |
|  | Min |  | 0.33 | | | 1 | | 2.67 | |  |  |  | |
|  | Max |  | 99.5 | | | 29.67 | | 31.33 | |  |  |  | |
|  | Std Dev |  | 46.65 | | | 12.28 | | 11.96 | |  |  |  | |
| ESXB | N |  | 5 | | | 5 | | 5 | | 0.911 | |  | |
|  | Mean |  | 19.07 | | | 33.26 | | 14.53 | |  |  |  | |
|  | Median |  | 20.5 | | | 7.33 | | 6.33 | |  |  |  | |
|  | Min |  | 0.33 | | | 0 | | 1 | |  |  |  | |
|  | Max |  | 42 | | | 122.33 | | 48 | |  |  |  | |
|  | Std Dev |  | 16.47 | | | 51.98 | | 19.34 | |  |  |  | |
| ADK | N |  | 5 | | | 5 | | 5 | | 0.651 | |  | |
|  | Mean |  | 233.9 | | | 38.6 | | 57.73 | |  |  |  | |
|  | Median |  | 31.5 | | | 5 | | 3 | |  |  |  | |
|  | Min |  | 1 | | | 0 | | 1.33 | |  |  |  | |
|  | Max |  | 1070.5 | | | 137.67 | | 266 | |  |  |  | |
|  | Std Dev |  | 467.88 | | | 58.5 | | 116.59 | |  |  |  | |

**Supplemental Table 8:** Antigen Conservation across Mycobacterial Species

| **Antigen** | **Species** | **% Identify** |
| --- | --- | --- |
| **AG85B** | ***M. bovis*** | **100%** |
|  | ***M. kansasaii*** | **90%** |
|  | ***M. avium*** | **86%** |
|  | ***M. intracellulare*** | **87%** |
|  | ***M. ulcerans*** | **89%** |
|  | ***M. marinum*** | **89%** |
|  | ***M. abscessus*** | **64%** |
|  | ***M. chelonae*** | **65%** |
| **PPE68** | ***M. bovis*** | **100%** |
|  | ***M. kansasaii*** | **77%** |
|  | ***M. avium*** | **51%** |
|  | ***M. intracellulare*** | **50%** |
|  | ***M. ulcerans*** | **60%** |
|  | ***M. marinum*** | **74%** |
|  | ***M. abscessus*** | **50%** |
|  | ***M. chelonae*** | **49%** |
| **ESXA (ESAT-6*)** | ***M. bovis **** | **100%** |
|  | ***M. kansasaii*** | **98%** |
|  | ***M. avium*** | **NA** |
|  | ***M. intracellulare*** | **33%** |
|  | ***M. ulcerans*** | **92%** |
|  | ***M. marinum*** | **91%** |
|  | ***M. abscessus*** | **48%** |
|  | ***M. chelonae*** | **33%** |
| **ESXB (CFP-10*)** | ***M. bovis **** | **100%** |
|  | ***M. kansasaii*** | **95%** |
|  | ***M. avium*** | **NA** |
|  | ***M. intracellulare*** | **NA** |
|  | ***M. ulcerans*** | **95%** |
|  | ***M. marinum*** | **97%** |
|  | ***M. abscessus*** | **40%** |
|  | ***M. chelonae*** | **NA** |
| **P9WKF5 (ADK)** | ***M. bovis*** | **100%** |
|  | ***M. kansasaii*** | **95%** |
|  | ***M. avium*** | **88%** |
|  | ***M. intracellulare*** | **87%** |
|  | ***M. ulcerans*** | **87%** |
|  | ***M. marinum*** | **86%** |
|  | ***M. abscessus*** | **71%** |
|  | ***M. chelonae*** | **54%** |

***ESXA and ESXB are deleted in BCG. NA= not available**

**Supplemental Figure Legend:**

**Supplemental Figure 1:** Gating strategy for surface staining flow cytometry. CD14/CD19 are combined for exclusion gating. LD = live/dead

**Supplemental Figure 2:** Gating strategy for intracellular flow cytometry. LD = live/dead.

**Supplemental Figure 3:** Cytokine production of MSTs from healthy donors following peptide restimulation demonstrated production of IL-13, Interferon-gamma, GM-CSF, MIP-1b, and TNFα. In MSTs from patients with PID, two of three tested samples only showed cytokine production with staphylococcal enterotoxin B (SEB) stimulation, and IL-8 production in a patient with IL-12R deficiency. Hu=human.

**Supplemental Figure 4**: Example Flow cytometry plots of MSTs derived from donors 2 and 5 demonstrating comparative perforin expression in CD4+ and CD8+ T-cells. Minor CD8+ populations express perforin at baseline, whereas perforin expression is minimal in CD4+ populations.

**Supplemental Figure 5**: Flow cytometry of MSTs derived from donors 9, 10 and a patient with NFKB1 haploinsufficiency demonstrated moderate expression of co-inhibitory receptors LAG3 and TIM3 in stimulated and unstimulated CD4+ and CD8+ T cells. GD2 chimeric antigen receptor-tranduced T-cells comparatively showed higher LAG3 and Tim3 expression at baseline and following stimulation.

**Supplemental Figure 6**: Surface immunophenotyping of MSTs produced using pepmix, sensitin, and lysate showed minimal differences in T cell subsets for the different growth conditions. The expanded cells were predominantly CD4+ effector memory T cells (CD4+/CD45RO+/CCR7-), with smaller central memory population (CD4/CD45RO^+^/CCR7^+^/CD62L^+^).

**Supplemental Figure 7**: Analysis of identified T cell epitopes from AG85B showed moderate to high conservation across mycobacterial species.

**Supplemental Figure 8**: Analysis of identified T cell epitopes from ESXB showed low to moderate conservation across mycobacterial species.
